# A Novel Hybrid Harris Hawk-Arithmetic Optimization Algorithm for Industrial Wireless Mesh Networks

**DOI:** 10.3390/s23136224

**Published:** 2023-07-07

**Authors:** P. Arun Mozhi Devan, Rosdiazli Ibrahim, Madiah Omar, Kishore Bingi, Hakim Abdulrab

**Affiliations:** 1 Department of Electrical and Electronic Engineering, Universiti Teknologi PETRONAS, Seri Iskandar 32610, Malaysia; arun.selvam@utp.edu.my (P.A.M.D.); rosdiazli@utp.edu.my (R.I.); hakim_19001004@utp.edu.my (H.A.); 2 Department of Chemical Engineering, Universiti Teknologi PETRONAS, Seri Iskandar 32610, Malaysia; madiah.omar@utp.edu.my

**Keywords:** Arithmetic Optimization Algorithm, Fractional-order Predictive PI, Harris Hawks Optimization, industrial wireless mesh networks, optimal node placement, pressure process, real-time control

## Abstract

A novel hybrid Harris Hawk-Arithmetic Optimization Algorithm (HHAOA) for optimizing the Industrial Wireless Mesh Networks (WMNs) and real-time pressure process control was proposed in this research article. The proposed algorithm uses inspiration from Harris Hawk Optimization and the Arithmetic Optimization Algorithm to improve position relocation problems, premature convergence, and the poor accuracy the existing techniques face. The HHAOA algorithm was evaluated on various benchmark functions and compared with other optimization algorithms, namely Arithmetic Optimization Algorithm, Moth Flame Optimization, Sine Cosine Algorithm, Grey Wolf Optimization, and Harris Hawk Optimization. The proposed algorithm was also applied to a real-world industrial wireless mesh network simulation and experimentation on the real-time pressure process control system. All the results demonstrate that the HHAOA algorithm outperforms different algorithms regarding mean, standard deviation, convergence speed, accuracy, and robustness and improves client router connectivity and network congestion with a 31.7% reduction in Wireless Mesh Network routers. In the real-time pressure process, the HHAOA optimized Fractional-order Predictive PI (FOPPI) Controller produced a robust and smoother control signal leading to minimal peak overshoot and an average of a 53.244% faster settling. Based on the results, the algorithm enhanced the efficiency and reliability of industrial wireless networks and real-time pressure process control systems, which are critical for industrial automation and control applications.

## 1. Introduction

Network Control Systems (NCS) have become increasingly popular in mining, process, and manufacturing industries that produce various goods such as food and beverages, different metals, multiple chemicals, pulp and paper, automobiles, textiles, crude oil refineries, and power generation plants [1]. NCS monitors and controls field instruments effectively, ensuring efficient and accurate operations [2]. In recent times, WMN has gained widespread acceptance in the industry, particularly with the wide acceptance of different wireless networking protocols, namely WIA-PA, Zigbee, WirelessHART, Bluetooth, and ISA 100 wireless. One of the primary advantages of these wireless standards is eliminating clunky cabling—a costly and time-consuming process to repair and maintain [3,4]. Additionally, WMN enables the expansion of network capability to the regions where it is difficult or impossible to install the wired cables, thus making monitoring and controlling operations in remote locations possible. Moreover, using WMNs increases data reliability, reduces data loss or interference, and offers improved security measures, protecting sensitive data from unauthorized access or theft [5]. It is essential to have a thorough knowledge of several factors such as transmission power, network topology, and wireless node density. Ignoring these factors while deploying WMN routers can lead to inadequate client coverage and connectivity, substandard network range, control-loop failures, and transmission loss [6]. It is crucial to consider the technical constraints of the deployment location and the basic topology before deploying wireless mesh routers. These constraints include factors such as radio signal strength, frequency range, and interference from other wireless networks [7,8].

WMNs offer many benefits for all the process control and automation industries [9]. However, some issues must be addressed to enhance the network’s performance, including network coverage, router connectivity, network traffic, compatibility, data security, etc. While comparing the outdoor regions, the indoor area is challenging while implementing the WMN for process monitoring and control because of the stochastic interferences [10]. In some outdoor areas, installations require fewer clients than in indoor regions. Furthermore, WMN can be implemented in multiple places with the availability of various control services in indoor and outdoor conditions. Determining the appropriate location for installing WMN routers for real-world applications is crucial. Therefore, optimization is essential for ensuring reliable and efficient WMN performance [11]. Optimization involves fine-tuning various parameters, such as router placement, transmission power, network topology, and routing protocols, to achieve the best possible network performance [12]. Optimizing these parameters allows the network to ensure that the WMN provides seamless coverage, reliable connectivity, and low latency, even in challenging industrial environments. Additionally, optimizing industrial WMNs can help improve network scalability and reduce maintenance costs [13].

At the same time, a feedback controller is essential in WMNs to ensure the system’s stability. In addition, the feedback controllers will help the system to maintain stable and accurate control over the process variables [14]. However, to achieve optimal performance, the controller must be optimized based on the system characteristics, process model, and external disturbances of the system it controls. Optimization techniques such as trial and error, Ziegler–Nichols, and the Cohen–Coon methods are commonly used to optimize feedback controllers. These conventional techniques involve adjusting the controller’s parameters to find the optimal values that result in suboptimal, unstable, and ineffective control. Thus, many researchers proposed using metaheuristic-based techniques to optimize the controller parameters for different applications [15,16,17,18,19,20].

The population-based evolutionary algorithms use mutations and crossovers, which produce new solutions using nature-inspired concepts from evolution. Swarm-based optimization techniques also employ these procedures [21]. However, they utilize two core processes for organizing initial swarms: scattering and intensifying them to specific high-potential regions [22]. Simultaneously, it should be noted that the No Free Lunch theorem affirms that no such existing metaheuristic optimization technique can be employed to solve every category of the existing issues in simulation or real-time conditions. In addition, this theorem highlights the need for a thorough understanding of the issue and the appropriate optimization technique selection to achieve optimal results [23]. Thus, a brief review of the Harris Hawk Optimization (HHO) and Arithmetic Optimization Algorithm (AOA) for different types of applications was carried out and is presented in the following sections for identifying the research gap for the proposed methodology.

### 1.1. Harris Hawk Optimization

Initial development of the Harris Hawk Optimization, a swarm-based optimization technique, was proposed by Heidari et al. [24]. This method is motivated by the cooperation and hunting strategies of Harris hawks in nature, which involve team action and reaction, as well as prey evasion. The main objective of HHO is to discover solutions to single-objective problems. The HHO method uses hawks’ chasing actions as search agents and the prey as the best position. This approach has the potential to solve a variety of real-world optimization problems, such as engineering design, pattern and speech recognition, manufacturing optimization, bio-mechanical engineering, power production quality, feature selection, and medical image segmentation [25,26,27,28,29]. As with other metaheuristic techniques, HHO uses exploration and exploitation phases to identify and catch the prey. However, the HHO differs from others in the exploitation phase, where it catches the prey based on soft and hard besiege and rapid dives on soft and hard besiege movements. Detailed information on the HHO algorithm can be found in these articles [24,25,28]. As a result of the benefits provided by HHO, there has been a significant increase in HHO research, with the number of studies continuing to grow rapidly to numerous applications and creating new HHO variations.

The authors of [30] aimed to accelerate the convergence of the HHO by proposing an improved version of the algorithm that employs two strategies. The first strategy involves improving the exploration phase of HHO by incorporating opposition-based learning and logarithmic spiral techniques. The second strategy combines the Modify Rosenbrock method to enhance the HHO’s local searching capability and convergence rate. The authors tested their algorithm by conducting experiments on 23 standard benchmark functions. They also compared their results with various state-of-the-art metaheuristic algorithms. In [31], two different variants of the HHO, namely binary HHO and quadratic binary HHO, were used to improve the feature selection problem at various data classification conditions. Binary HHO has a transfer function that can be either S-shaped or V-shaped, effectively converting a continuous variable into a binary one. The quadratic binary HHO is designed to improve the binary HHO by incorporating a binary quadratic model.

Dokeroglu et al. [32] developed a binary variant of the multi-objective HHO approach to address a classification task. Their novel discrete operators for exploitation (besieging) and exploration (perching) were introduced. Their study employed four machine learning methods applied to a COVID-19 dataset for binary classification problems to increase prediction accuracy. Reference [33] proposed a novel optimization algorithm, called the modified HHO, to reconfigure photovoltaic modules and disperse shadow regularly to increase the power generated. The algorithm was tested on different shade patterns with 9 × 9, 6 × 4, and 6 × 20 photovoltaic arrays based on the available simulation data. The outcomes were analyzed and compared with varying techniques of reconfiguration based on metrics such as mismatch power, fill factor, power loss, and power enhancement. Hussain et al. designed an improved variant of the HHO named long-term memory HHO that enhances the diversity of search agents by maintaining a broad search region for the exploration phase resulting in better convergence results. The proposed technique uses different engineering optimization test problems, including power flow optimization for the power generation systems [34].

The improved HHO algorithm, as introduced by Houssein et al. [35], addresses the challenge of optimizing large-scale wireless sensor networks. The approach involves utilizing Prim’s shortest path algorithm to establish minimum transmission paths from the sink node to all other sensor nodes in order to reconstruct the network. The researchers have proposed energy-efficient wireless sensor networks using a hybrid Harris hawk-salp swarm optimization algorithm in [36]. The approach uses a hybrid optimization algorithm combining the HHO and salp swarm techniques to optimize the network’s energy consumption. Additionally, a mobile sink strategy reduces energy consumption by allowing the destination node to move toward the sensor nodes instead of the other way around. For PID controller optimization, researchers used HHO to control the speed of the DC motor [37]. Using the HHO algorithm for tuning the controllers improves performance in terms of steady-state error, rise time, overshoot, and settling time, resulting in better efficiency, accuracy, and stability of the motor control system. An overall detailed review of the HHO literature is given in Table 1.

### 1.2. Arithmetic Optimization Algorithm

The AOA is a recently designed mathematics-inspired metaheuristic optimization technique that draws inspiration from the distribution behavioral nature of arithmetic functions from the mathematics proposed by Abualigah et al. [40]. It uses basic arithmetic operations, such as addition, subtraction, multiplication, and division, as the primary operator to explore the desired objective function and the optimal solution. AOA is also a population-based optimization technique, a set of solutions, called individuals, are generated randomly and evaluated based on their fitness concerning the considered problem. The best-fit individuals are chosen and subjected to various mathematical operations to generate new individuals based on the desired objective values in both phases. These new individuals are then evaluated for their fitness and the process is repeated until the desired optimization level is reached [41].

A key advantage of AOA is that it can prevent getting stuck in local minima, a common issue many optimization algorithms face. AOA achieves this by employing a mechanism called elitism, which ensures that the best solutions in the population are always preserved and passed over to the next generation, allowing the algorithm to explore different areas of the solution and converge to the global minimum efficiently. Premkumar et al. [42] proposed a newly developed approach called Multi-Objective AOA (MOAOA) to solve real-world constrained multi-objective optimization problems. The latest version of the AOA algorithm has been improved by adding two new functions—elitist non-dominance sorting and a crowding distance-based mechanism. This new algorithm has been tested on 35 optimization problems with constraints and five test problems without constraints. Its effectiveness has been compared with four other advanced multi-objective optimization techniques. Additional performance metrics are also considered to evaluate the MOAOA for higher accuracy and effective convergence, in which MOAOA achieved superior performance.

In [43,44,45], the researchers developed an enhanced form of artificial neural network which uses a two-step process to identify, locate, and measure the extent of damage in plate structures made of functionally graded materials, petroleum products, and other electrical systems. In the first stage, a damage indicator based on the frequency response function is utilized to forecast which components of the material have been affected. In the second stage, the networks are employed to quantify the damage and it eliminates healthy elements from the numerical model and utilizes information from the defective components to estimate the degree of damage. Abualigah et al. [46] proposed a new method for various multilevel thresholding in data analysis and image segmentation using the AOA and differential evolution technique. The DAOA algorithm was assessed using traditional examination images from nature and CT COVID-19 images. The accuracy of the segmented images was measured using the peak signal-to-noise ratio and structural similarity index test.

Zheng et al. [47] developed a new hybrid algorithm called DESMAOA, combining two meta-heuristic algorithms—the Slime Mold Algorithm (SMA) and the AOA. The DESMAOA algorithm was designed to improve the optimization capability of the existing techniques. The SMAOA algorithm is first used to improve the SMA algorithm, and then two strategies from SMA and AOA are combined to create the DESMAOA. In [48], a new method is developed to control an automatic voltage regulator using a robust Model Predictive Controller (MPC). The suggested approach was crafted to address the challenge of uncertain automatic voltage regulation parameters. Frequency domain conditions are obtained through the Hermite–Biehler theorem to ensure stability in the face of perturbations. The tuning of MPC parameters is performed using the AOA technique while accommodating stability constraints. A time-domain objective is established to optimize the voltage regulator’s performance by minimizing voltage peak overshoot and fastening the process settling.

In [49], the authors introduced a forced switching mechanism for AOA termed IAOA that helps search agents to switch between various local optima. The effectiveness of the IAOA was tested on multiple benchmark functions and real-world test problems and the results showed that it outperforms other optimization algorithms in most cases. Different types of metaheuristic optimization were compared for the various engineering problems in [50]. The researchers proposed an enhanced version of the AOA called nAOA. The nAOA technique uses logarithmic and exponential mathematical operators to improve algorithm performance. The overall summary of the AOA technique is given in Table 2.

Some of the main contributions of this research article are listed as follows:

1. The primary motivation of HHAOA is to solve the problem of placing the routers optimally to achieve adequate coverage and connectivity, along with finding the best parameter for the Fractional-Order Predictive PI (FOPPI) controller.
2. The proposed HHAOA technique makes use of all the arithmetic operator combinations, enabling smooth and efficient relocations between local minima without getting stuck. This approach effectively minimizes computational complexity, resulting in a more streamlined process outcome.
3. To achieve a better convergence rate and reach the desired optimal solution location, the proposed optimization was simulated and validated using 33 different benchmark functions.
4. The proposed technique was implemented to enhance nodes’ placement and mitigate congestion in wireless mesh networks (WMNs). The results reveal that the HHAOA technique is highly effective in reducing the number of node placements, leading to significant cost savings and improved network congestion compared to alternative algorithms.
5. The HHAOA-optimized FOPPI controller was implemented on a real-time pressure process plant to validate our proposed optimization algorithm, and the results show a better performance than conventional controllers.

## 2. Proposed Hybrid Harris Hawks-Arithmetic Optimization Algorithm

The primary focus of this study was developing a hybrid HHAOA technique to enhance the coverage and connectivity of a WMN, minimize network congestion, and optimize parameters for the FOPPI controller [54]. The proposed technique hybridizes the HHO and AOA techniques. Despite the impressive capabilities of HHO and AOA, some drawbacks need to be addressed, including the risk of premature convergence, becoming trapped in multiple local optima, and its phase-switching mechanism. The proposed HHAOA approach improves convergence behavior, the position-switching mechanism, and solution quality. When implementing the hybrid method for searching, the process becomes notably more thorough and effective. This is due to the ability to navigate throughout the desired search area and avoid becoming trapped in local optima. As a result, a diverse range of potential solutions can be generated, increasing the likelihood of finding the optimal outcome. Furthermore, the HHAOA will be used to optimize the placement of WMN routers via simulation. Additionally, real-time experiment experimentation on the pressure process plant using the HHAOA-optimized FOPPI controller was compared with traditional techniques.

### 2.1. Proposed Hierarchical Structure

**Figure 1:**
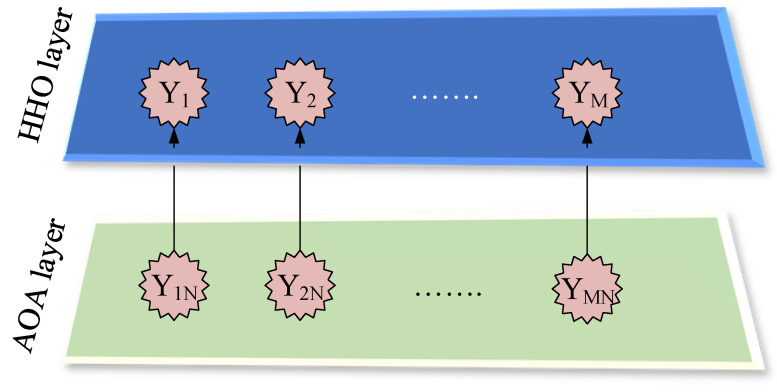
Hierarchy form of the proposed HHAOA optimization algorithm.

The proposed HHAOA hierarchical structure is depicted in Figure 1. The system comprises a top layer (primary layer) with *M* HHO search agents and a bottom layer (secondary layer) with groups containing an *N* AOA population. The AOA execution in the bottom layer initiates the process of updating the search agents’ positions. In order to find the most optimal solution, it is crucial to ensure that the positions of all search agents in the upper layer are updated with the best solution discovered by the corresponding group in the lower layer. New equations can be formulated for both the exploitation and exploration phases, leading to an even more effective solution. This approach allows for a comprehensive problem analysis and significantly improves the overall outcome.

### 2.2. Exploration

This section proposes an exploration mechanism for hybridizing the HHO and AOA algorithms. The algorithm draws inspiration from the hunting behavior of Harris hawks, capitalizing on their exceptional eyesight to effectively track and recognize prey. However, the prey may not be immediately visible in some cases, prompting the hawks to patiently wait, observe, and monitor the objective location for several hours until detecting a potential target.

The algorithm expertly utilizes Harris hawks to represent potential solutions, with the ultimate goal of comparing the best solution to the ideal outcome. The algorithm flawlessly imitates the hawks’ actions by strategically placing them in different locations and using various approaches until the desired solution is found. Each strategy is meticulously employed in equal measure to ensure maximum efficiency. The hawks rest closer to the prey (rabbit) based on the positions of other family members during q<0.5. When the condition is met with q≥0.5, the hawks will randomly perch on tall trees within the range of their family members. Instead of selecting the random tall trees, the best position nearest to the rabbit will be identified using the AOA algorithm, which will further enhance the chances of reaching and identifying the target much faster. Therefore, the exploration strategy of the proposed HHAOA is obtained using the equation given below.

(1) $$Y_{j+1}^{i}=\left\{\begin{array}{c} y_{\mathrm{rand}\;}-r_{1}|y_{\mathrm{rand}\;}-2r_{2}(y_{j}÷\left(\mathrm{MOP}+\epsilon \right)\times ((UB_{j}\\ -LB_{j})\times \;\mu +LB_{j})|,q\geq 0.5\;\&\;c\geq 0.5\\ y_{\mathrm{rand}}-r_{1}|y_{\mathrm{rand}}-2r_{2}(y_{j}\times \mathrm{MOP}\times (\left(UB_{j}-LB_{j}\right)\\ \times \;\mu +LB_{j})|,q\geq 0.5\;\&\;c<0.5\\ y_{B}-y_{m}-r_{3}\left[LB_{j}+r_{4}\left(UB_{j}-LB_{j}\right)\right],q<0.5, \end{array}\right.\;$$

where $Y_{i}^{j+1}$ describes the location of *t*-th solution in the top layer (HHO) corresponding to the *j*-th search solution in the AOA layer. *j* represents the current iteration of the algorithm. $r_{1}$, $r_{2}$, $r_{3}$, $r_{4}$, *c*, and *q* are the random numbers that lies in the span of [0,1]. $y_{rand}$, $y_{B}$, $y_{m}$, UB, LB, MOP are the random hawk selected, the best location obtained so far in the present iteration, average mean of the hawk’s position, upper and lower bound ranges of the variables, and the math-optimizer probability coefficient. The MOP and average mean of the hawk’s location values are calculated based on the equation given below.

(2) $$\mathrm{MOP}\left(j\right)=1-{\left(\frac{t}{T}\right)}^{1/\alpha }\;$$

(3) $$y_{m}\left(j\right)=\frac{1}{N}\sum_{j=1}^{N}y_{i}\left(j\right),\;$$

where α is a crucial parameter factor that determines the precision of the exploitation phase, *t*, and *T* represent the current and the highest possible number of iterations, respectively. The position of every hawk in iteration j is represented by $y_{i}\left(t\right)$, and the total number of hawks is denoted by *N*.

### 2.3. Transition Stage

The HHO algorithm can alternate between exploration and exploitation modes depending on the amount of energy the prey has left while attempting to escape. The prey’s energy decreases notably during its escape. This energy is determined by using the equation below:

(4) $$E=2E_{0}\left(1-\frac{t}{T}\right),\;t=\left\{1,2,3,\ldots ,T\right\}.$$

The escaping energy of the prey is represented as E and the maximum number of iterations is represented by T with the current iteration being represented as t. Additionally, $E_{0}$ indicates the initial energy state of the prey, which randomly changes within the interval of (−1,1) at each iteration in HHO. When $E_{0}$ decreases from 0 to −1, the prey is physically exhausted, while an increase in $E_{0}$ from 0 to 1 demonstrates that the prey is becoming more powerful. During the iterations, the escaping energy (E) gradually decreases. When the escaping energy is |E| ≥1, the hawks explore various locations to search for the prey, thereby performing the exploration phase. Conversely, when |E| < 1, the algorithm focuses on exploiting the neighbourhood of the solutions.

### 2.4. Exploitation

During the attacking phase, Harris hawks employ a surprise pounce maneuver to take down their intended prey, which is identified in the previous stage. However, the prey may attempt to evade danger, resulting in various chasing styles observed in actual real-life scenarios.

Based on the prey’s fleeing behavior and the Harris hawks’ pursuit strategies, the HHO proposes four potential approaches to simulate the attacking phase. As preys always try to escape threatening situations, they have a chance (r) of successfully escaping (r<0.5) or not (r≥0.5) before the surprise pounce. Hawks employ a hunting technique known as hard or soft encirclement to capture their prey which involves surrounding the prey from various angles, considering its energy level. The hawks gradually move closer to their prey to increase their chances of a successful surprise attack in practical settings. As the fleeing prey loses energy over time, the hawks intensify the besiege process to capture the exhausted prey effortlessly. Parameter E models this strategy and enables the HHO to alternate between soft and hard besiege processes. Similar to the exploration, the AOA will reach the solution (prey) closer in this phase.

#### 2.4.1. Soft Besiege

The rabbit retains sufficient energy to flee through random and deceptive jumps during r≥0.5 and |E| ≥0.5. However, its efforts prove to be unsuccessful, and the Harris hawks gradually encircle it, causing the rabbit to become increasingly tired before ultimately launching a surprise attack. The following Equation (5) represents this surprise attack of HHAOA:

(5) $$Y_{j+1}^{i}=\left\{\begin{array}{c} y_{B}-\left[F_{A}\right]-E|2\left(1-r_{2}\right)y_{B}-\left[F_{A}\right]|,c<0.5,\\ y_{B}-\left[F_{B}\right]-E|2\left(1-r_{2}\right)y_{B}-\left[F_{B}\right]|,c\geq 0.5, \end{array}\right.\;$$

where

$F_{A}=y_{j}-\mathrm{MOP}\times \left(\left({UB}_{j}-{\mathrm{LB}}_{j}\right)\times \mu +{LB}_{j}\right)$

$F_{B}=y_{j}+\mathrm{MOP}\times \left(\left({UB}_{j}-{\mathrm{LB}}_{j}\right)\times \mu +{LB}_{j}\right)$.

#### 2.4.2. Soft Besiege with Progressive Rapid Dives

When the rabbit’s energy is sufficient (|E| ≥0.5) for successful escape and the hawk’s movement is rapid (r < 0.5), a gentle ambush is carried out before the surprise attack. This approach is more intelligent than the previous method. To mathematically model the prey’s escape patterns and the predator’s leapfrog movements, the HHAOA algorithm utilizes the idea of incorporating the Levy flight (LF) pattern. LF simulates the erratic zigzag movements of prey (especially rabbits) in the fleeing phase and hawk’s irregular, sudden, and rapid dives as they approach the targeted prey. The LF mechanism used here is given below.

(6) $$LF\left(x\right)=\frac{\mu \times \delta }{{\left|\nu \right|}^{\frac{1}{\zeta }}}\times 0.01,\delta ={\left(\frac{\Gamma \left(1+\zeta \right)\times \sin\left(\frac{\pi \beta }{2}\right)}{\Gamma \left(\frac{1+\zeta }{2}\right)\times \zeta \times 2^{\left(\frac{\zeta -1}{2}\right)}}\right)}^{\frac{1}{\zeta }},$$

where μ and ν are random numbers that lie in the range of inside [0,1], ζ is a constant value that is set at 1.5.

During the soft besiege, hawks perform multiple rapid dives around the rabbit, constantly adjusting their position and direction to match the deceptive movements of the prey. This mechanism is also observed in other competitive situations in nature. The movement of the hawks in this phase is obtained using the following position update rule given in Equation (7).

(7) $$Y_{j+1}^{i}=\left\{\begin{array}{c} Z\;\;\mathrm{if}\;\;F\left(Z\right)<F\left(y_{j}\right)\&\\ y_{j}=\left\{\begin{array}{c} F_{A},c<0.5\\ F_{B},\;c\geq 0.5 \end{array}\right.\\ X\;if\;F\left(X\right)<F\left(y_{j}\right)\&\\ y_{j}=\left\{\begin{array}{c} F_{A},c<0.5\\ F_{B},\;c\geq 0.5, \end{array}\right. \end{array}\right.$$

where

Z=X+S×LF(D)

$X=y_{B}-E|Jy_{B}-y_{m}|;J=2-2r_{2}$

*S* = Random vector in the dimension D (1×D).

#### 2.4.3. Hard Besiege

When the conditions meet r≥0.5 and |E| <0.5, the rabbit will become exhausted and have less energy to flee. As a result, the Harris hawks will attempt to capture the rabbit by surrounding it aggressively and launching surprise attacks. The following equation can describe this intense attack phase.

(8) $$Y_{j+1}^{i}=\left\{\begin{array}{c} y_{B}-E|y_{B}-[y_{j}-\mathrm{MOP}\times \;(\left({UB}_{j}-{\mathrm{LB}}_{j}\right)\times \\ \mu +{LB}_{j})]|,\;c<0.5\\ y_{B}-E|y_{B}-[y_{j}+\mathrm{MOP}\times \;(\left({UB}_{j}-{\mathrm{LB}}_{j}\right)\times \\ \mu +{LB}_{j})]|,\;c\geq 0.5. \end{array}\right.$$

#### 2.4.4. Hard Besiege with Progressive Rapid Dives

If |E| <0.5 and *r* <0.5, the rabbit lacks the necessary energy to flee and must resort to a sudden, aggressive dive technique to catch its prey off guard. This maneuver places the prey in a position similar to a soft dive. Still, the hawks work to minimize the distance between themselves and the prey’s average location while attempting to escape. The movement of this phase is obtained as follows:

(9) $$Y_{j+1}^{i}=\left\{\begin{array}{c} Z\;\;\mathrm{if}\;\;F\left(Z\right)<F\left(y_{j}\right)\&\\ y_{j}=\left\{\begin{array}{c} F_{A},c<0.5\\ F_{B},\;c\geq 0.5 \end{array}\right.\\ X\;if\;F\left(X\right)<F\left(y_{j}\right)\&\\ y_{j}=\left\{\begin{array}{c} F_{A},c<0.5\\ F_{B},\;c\geq 0.5 \end{array}\right. \end{array}\right.$$

where,

Z=X+S×LF(D)
$X=y_{B}-E|Jy_{B}-y_{j}|$.

The HHAOA proposal consists of two distinct phases: exploration and exploitation. The initial phase is responsible for finding a new position to enhance the optimization process. At the same time, the latter stage utilizes four different strategies to maximize the position update to converge effectively. Detailed information about when to switch between these two phases is presented in Table 3.

### 2.5. Pseudocode of Proposed Algorithm

**Figure 2:**
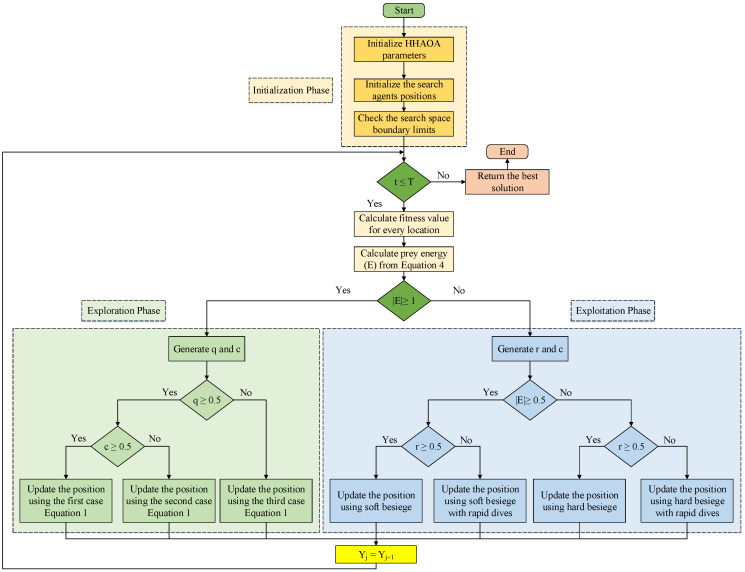
Flowchart of the proposed HHAOA technique.

The application of HHAOA for the industrial wireless mesh networks (WMN) and FOPPI controller parameter optimization is comprehensively explained in the pseudocode for the proposed technique presented in Algorithm 1. The respective implementation of the HHAOA using the flowchart is illustrated in Figure 2.

### 2.6. Algorithm Complexity

It is crucial to note that the complexity of the proposed HHAOA algorithm is heavily influenced by three critical processes: initialization of the transition stage, fitness function evaluation, and position updating of Hawks. The initialization process has a complexity of C(n) when there are n hawks. Updating the location vector of all hawks involves searching for the optimal location, which can be complex. This involves C(T × n) + C(T × n × D) operations, where T represents the maximum number of iterations and D is the dimension of the current particular problem in hand. Therefore, it is imperative to understand that the computational complexity of HHAOA can be expressed as C(n × (T + TD + 1)).
**Algorithm 1** Pseudocode of HHAOA technique.**Input:** Random WMN routers positions and manually calculated FOPPI controller parameters.**Output:** Optimal WMN connection and FOPPI parameters1:Initialize the search agents positions $y_{j}$ (*j* = 1, 2, 3, ..., N)2:Check search space boundary and initiate the t≤T case3:**while** (t≤ T) **do**4:    Attain the initial solution $y_{B}$5:    **for** (All hawks ($y_{j}$)) **do**6:        Amend the position update7:        Use (4) to update the E8:        **Phase: Exploration**9:        **if** |E| ≥1 & q≥0.5 **then**10:           **if** c≥0.5 **then**11:               Update the solution using Equation (1) condition 112:           **else if** c<0.5 **then**13:               Update the solution using Equation (1) condition 214:           **end if**15:       **else if** |E| ≥1 & q<0.5 **then**16:           Update the solution using Equation (1) condition 317:        **end if**18:        **Phase: Exploitation**19:        **if** |E| < 1 **then**20:           **if** (|E|≥ 0.5 and r ≥ 0.5) **then**21:               **if** c<0.5 **then**22:                   Solution update using Equation (5) condition 123:               **else if** c≥0.5 **then**24:                   Solution update using Equation (5) condition 225:               **end if**26:           **else if** (|E|≥ 0.5 and r < 0.5) **then**27:               **if** c<0.5 or c≥0.5 **then**28:                   Solution update using Equation (7) condition 129:               **else if** c<0.5 or c≥0.5 **then**30:                   Solution update using Equation (7) condition 231:               **end if**32:           **else if** (|E|<0.5 & r ≥ 0.5) **then**33:               **if** c<0.5 **then**34:                   Solution update using Equation (8) condition 135:               **else if** c≥0.5 **then**36:                   Solution update using Equation (8) condition 237:               **end if**38:           **else if** (|E|< 0.5 & r < 0.5) **then**39:               **if** c<0.5 or c≥0.5 **then**40:                   Solution update using Equation (9) condition 141:               **else if** c<0.5 or c≥0.5 **then**42:                   Solution update using Equation (9) condition 243:               **end if**44:           **end if**45:        **end if**46:    **end for**47:    *t* = *t* + 148:**end while** 49:**Return** $Y_{B}$ for optimal WMN connectivity with all the routers and optimal FOPPI controller parameters for real-time pressure process plant.

## 3. Problem Formulation

The main focus of this subsection is the implementation of the proposed HHAOA approach, which addresses the problem of optimal placement of WMN routers and finding the optimal FOPPI controller parameters. The effectiveness of the HHAOA method was compared against different metaheuristic optimization techniques, such as AOA, MFO, SCA, GWO, WOA, and HHO. The benchmark algorithms and the proposed method were evaluated using MATLAB software and the results were analyzed based on the original positions of the clients generated using the Atarraya simulator [25].

### 3.1. Industrial Wireless Mesh Networks

Proper planning is essential when implementing a wireless mesh network to ensure the optimal placement of mesh routers. Determining the ideal locations and the number of routers needed to achieve complete coverage and connectivity is crucial. Our approach assumes that mesh clients remain stationary and their locations are predetermined, as the placement of mesh routers in an industrial environment depends on the clients’ locations. Despite this, finding the optimal placement of WMN routers in a timely and precise manner remains a computational challenge. To address this issue, we made certain assumptions about the placement of mesh routers in a wireless mesh network.

- The devices connected to the mesh network stay in one place within a desired 2D region;
- Each router in the network has the same range for transmitting signals (Identical transmission);
- The routers are connected based on their transmission range to ensure connectivity.

The location of mesh clients establishes the ideal positioning of mesh routers and it is represented by $M=\left\{L\left(p_{1},q_{1}\right),L\left(p_{2},q_{2}\right),L\left(p_{3},q_{3}\right),\cdots ,L\left(p_{n},q_{n}\right)\right\}$. It is crucial to remember that a network N may not be entirely linked, meaning it could consist of multiple distinct subgraphs. In order to improve the connectivity of the WMN, it is important to enlarge the largest subgraph in the network until it reaches its maximum capacity. A network is considered fully linked when all mesh routers are interconnected. The network coverage for the clients can be obtained using the following equation.

(10) $$\Omega \left(N\right)=\sum_{d=0}^{c}\Delta_{d}\;$$

where, $\Delta_{d}=\left\{\begin{array}{c} 1\;\mathrm{if}\;\mathrm{the}\;\mathrm{router}\;\mathrm{encloses}\;\mathrm{the}\;\mathrm{client}\;d\\ 0\;\mathrm{Otherwise}. \end{array}\right.$

Similarly, the network connectivity between the WMN routers will be measured using the expression below.

(11) $$\Phi \left(N\right)=\max_{d\in \{1,\ldots ,h\}}\left|N_{d}\right|.$$

The primary goal of the HHAOA is to enhance the efficiency of the WMN, aiming to maximize the network range and connection while minimizing the usage of mesh routers to alleviate network traffic. Moreover, when establishing the fitness function, the parameters Φ(N), representing the level of connectivity among routers, and Ω(N), stating the network coverage, were considered. The weighted sum technique is a straightforward approach for streamlining a multi-objective problem. By expertly combining each objective and assigning a user-determined weight, a single-objective problem is formed.

(12) $$F_{j}=\xi \cdot \left(1-\frac{\Phi (N)}{n}\right)+\left(1-\xi \right)\cdot \left(1-\frac{\Omega (N)}{m}\right);$$

where, the m and n are the numbers of mesh network clients and routers, respectively. ξ is a weight-adjusting coefficient parameter that lies in the range of (0,1). Furthermore, the metrics for the statistical comparison will be carried out in terms of mean, standard deviation (std.), and best and worst.

### 3.2. Pressure Process Control

**Figure 3:**
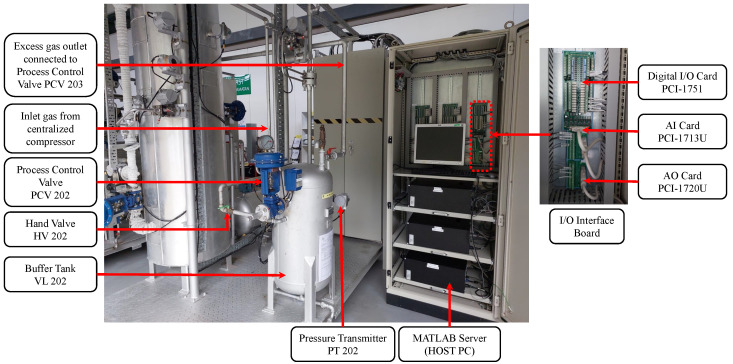
Real-time schematic of the pressure process plant.

Figure 3 illustrates the schematics of the real-time pressure process plant, which operates in real-time. The primary buffer tank, VL 202, was designed to withstand up to 10 Bar of pressure from the centralized air compression system that supplies air to it. The pressure inside the tank can be controlled using the hand valve, HV 202, while the process control valve, PCV 202, ensures that the pressure inside the tank is maintained at the desired level. The pressure transmitter, PT 202, is utilized to measure the pressure, which is then converted to digital voltage signals ranging from 0 to 5 V. These signals are sent to the pressure indicating controller PIC 202, which transmits the control signal to the host PC via I/O interface boards.

For safety purposes, there is an analog pressure gauge that displays pressure changes inside the tank. In case of an emergency, there is a hand-operated valve at the bottom of the buffer tank that releases compressed air from the VL 202 if the valve PCV 202 fails. The hand valve can also be used as an external disturbance injection channel during experimentation. The pressure inside the buffer tank is regulated by releasing excess air through an outlet on the top of the process tank, which is connected to another process control valve (PCV 203). This valve is kept at a 50% opening during the experiment to prevent excessive pressure build-up inside the VL 202. The host PC sends signals to the control valve actuator PCV 202 based on the set-point value.

**Figure 4:**
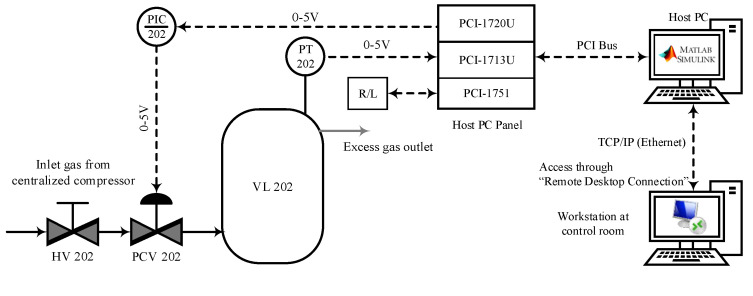
P&I diagram of the pressure process plant.

Figure 4 displays the piping and instrumentation diagram of the pressure process plant. The plant operates in “Remote Desktop Connection” mode for safety, controlling processes from the central control room. The communication between the mainframe PC and field devices, such as the control valve actuator, flow sensors, and pressure transmitter, is established with PCI cards. These cards provide isolation protection of 2500 V DC between the PCI bus outputs. The process plant’s analog input is received by a 32-channel analog input card called PCI-1713U. It has a 12-bit resolution and a sampling rate of 100 k samples. The PCI-1720U module, a crucial component of the PCI card, employs a high-quality 12-bit, 4-channel analog output port to transmit precise control signals to the host PC. Furthermore, the PCI-1751 card allows for remote control of the pressure process plant by facilitating data transmission of digital signals from the PT 202 to the host PC and vice versa, with 48 bits of parallel digital input/output.

Obtaining the transfer function of the pressure process plant is achievable through mathematical modeling using the open-loop response for the step input signal. By applying the characteristic equation of the first-order plus dead-time system, the final transfer function of the plant is equated as given below.

(13) $$G_{p}\left(s\right)=\frac{K}{1+Ts}e^{-sL_{p}}=\frac{0.866}{1+1.365s}e^{-s};\;$$

where, *K* is the process gain, $L_{p}$ represents system dead-time, and *T* is the system time constant.

**Figure 5:**
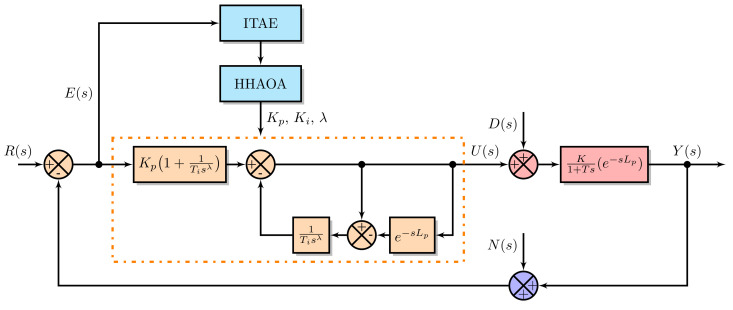
FOPPI controller tuning using HHAOA.

Fractional-order predictive PI controller is a dead-time compensating control mechanism proposed by Arun et al. to overcome the limitations of conventional PI controllers [54]. The FOPPI controller’s design is characterized by its simplicity and effectiveness, and it boasts the impressive capability to compensate for dead-time and is exceptionally adept at rejecting stochastic disturbances. The FOPPI controller integrates the dead-time compensating ability of the Smith predictor with the robust nature of fractional-order controllers. The FOPPI controller is particularly useful for non-linear, fast response, and sensitive applications such as pressure processes. The controller produces an adequate robust control signal unaffected by load changes or plant uncertainties. However, the controller parameters that are identified through analytical techniques may not be enough to create an effective control signal. This can cause problems with the performance of the plant. In order to overcome this issue, the proposed HHAOA and various optimization algorithms were used to obtain the optimal controller parameters. These parameters were then used to investigate the pressure process plant. The FOPPI control signal was generated using the equation provided below.

(14) $$u\left(s\right)=K_{p}\left(1+\frac{1}{T_{i}s^{\lambda }}\right)e\left(s\right)-\frac{1}{T_{i}s^{\lambda }}\left(1-e^{-sL_{p}}\right)u\left(s\right);\;$$

where, $K_{p}=\frac{1}{K}$. Let u(s) and e(s) represent the control and error signals, respectively. $K_{p}$ represents the proportional gain, $T_{i}$ denotes the integral time, λ represents the fractional-order integrator, and $L_{p}$ represents the process dead-time. The proposed HHAOA technique was used for obtaining the FOPPI controller parameters with the goal of minimizing the integral time absolute error (ITAE) value. The ITAE value was used as the objective function for all optimization algorithms. Figure 5 shows how the FOPPI controller was tuned using the HHAOA method. The ITAE value can be calculated using the following formula:

(15) $$\mathrm{ITAE}=\int_{0}^{\infty }t\left|e\left(t\right)\right|dt.$$

## 4. Results and Discussion

This section presents the benchmark functions and wireless mesh network simulation analysis in the first and second subsections. Lastly, the HHAOA-optimized FOPPI controller was experimented on with the pressure process plant. During the simulation of the benchmark functions, the Hawks population was kept at 100, and ran for 300 iterations, while in the WMN the simulation ran for 500 iterations, keeping the Hawks population constant with dimensions of 1000 m by 1000 m. The experimentation and simulation were conducted in the Intel(R) Xeon PC 3.10 GHz and 16.00 GB RAM using the MATLAB/Simulink software (2021a). Additionally, extra parameters for the algorithms are listed as min = 0.2, max = 1.0, convergence constant a=2, initial and escaping energies $E_{0}$ = [0 1] and *E* = [−1 1], α = 5, μ = 1.5, and $\epsilon =2.2204\times 10^{-16}$.

### 4.1. Performance Analysis on Benchmark Functions

A simulation analysis for 30 benchmark functions (refer to Table 4 and Table 5) using AOA, MFO, SCA, GWO, WOA, HHO, and the proposed HHAOA was conducted. The selected benchmark functions were independent of each other in multiple dimensions and had various local minima, global minima ($G_{m}$), different ranges, and diverse boundary values to ensure a reliable comparative analysis. Furthermore, the algorithms were comprehensively tested using multimodal and hybrid composition functions in single and multiple dimensions.

#### 4.1.1. Benchmark Functions

The algorithm’s accuracy was tested using unimodal functions (F1 to F7). As for multimodal functions, they contain multiple local minimum points, which serve as a measure of the algorithm’s exploration capacity. This capacity pertains to its ability to move seamlessly from local to global minima without getting stuck in a single position. Table 4 shows the F8 to F13 multimodal benchmark functions that have many local minima. The F14 to F21 functions also have multiple local minima with fixed dimensions. These functions help to evaluate the stability of optimization algorithms. It is necessary to mention that the first 13 benchmark functions have higher dimension values of 30, 100, 500, and 1000 to evaluate the proposed HHAOA.

**Figure 6:**
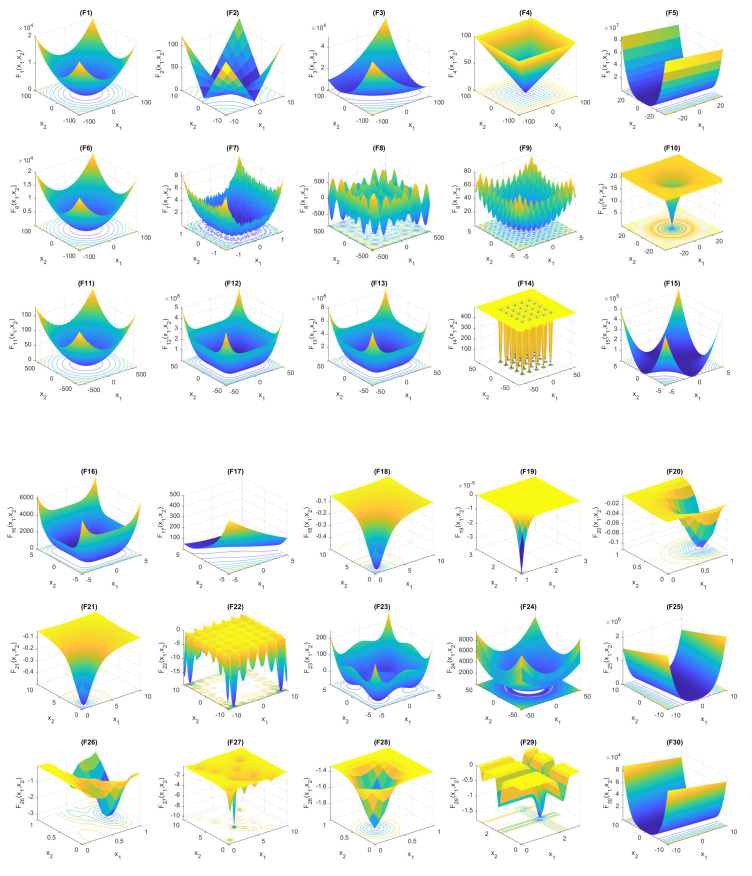
Benchmark functions search space plots.

The remaining functions, from F22 to F30, are hybrid functions as detailed in Table 5. While working with unimodal functions, finding the optimal solutions was comparatively effortless as they were conveniently located and easily reachable from the current location. The likelihood of being trapped in a particular search area is minimal. Finding the optimal position and movement between hybrid functions F19 and F33 is very difficult because there are many local minimum values to consider in the search space. Figure 6 displays the surface plots for all functions, providing a clear visualization of the search space needed to identify the global minimum for both single and multi-minima functions. Functions can be classified into different categories based on their surface area and shape. These categories include bowl-shaped, plate-shaped, and valley-shaped functions. There are also functions with single or multiple local minima. The latter type of function has a broad search space, wide range, numerous layers, and multi-dimensional features due to its multiple local minima values.

#### 4.1.2. Convergence Analysis

**Figure 7:**
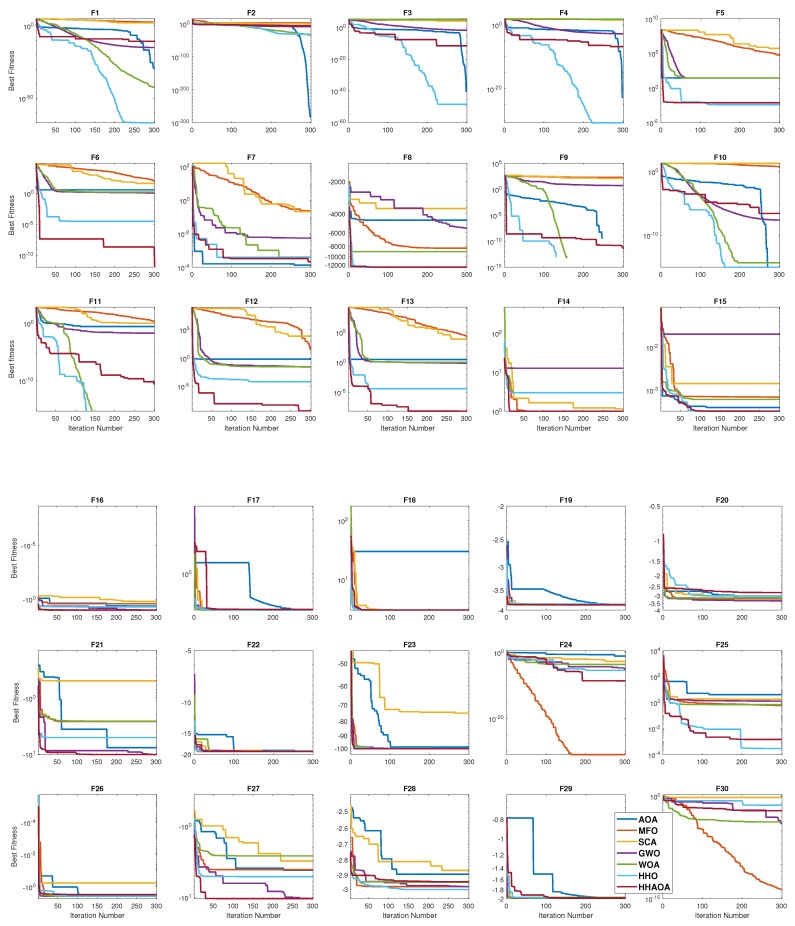
Convergence performance of all the benchmark functions.

The benchmark procedures were evaluated through 300 iterations, with 100 for the loop condition, to determine their convergence properties. Convergence, in this context, indicates the point at which an algorithm locates the smallest possible fitness value within the specified number of maximum iterations. Figure 7 illustrates the convergence analysis plot for all the benchmark functions in the presence of all the optimization algorithms.

In the unimodal functions (F1–F4), the conventional HHO had the fastest convergence in reaching the global minima. However, in the remaining functions, the proposed HHAOA relocated the positions faster, reaching the objective function in the first place. In this performance, the MFO had the major setback of exploring desired search regions, leading to inadequate performance and securing the last position. In the multimodal functions (F8–F13), using the hawk’s population effectively, the proposed HHAOA had a better transition phase from exploration to exploitation while noticing the convergence speed, which has a more significant speed of reaching the global minima with fewer iterations. In addition, the amplitude reduction rate is directly proportional to its convergence speed in the HHAOA performance in these functions. These results, in turn, illustrate the ability of HHAOA to focus more on the desired search locations during the iterations.

It is important to note that, in F9, many algorithms were unable to reach the desired value because of the existence of multiple minimum values. (see Figure 6). Still, the HHAOA explicitly performed well and had the best and faster convergence near the global minima. SCA and MFO had the least convergence speed in these functions, followed by GWO, WOA, and AOA, respectively. Notably, the conventional HHO had performance comparable to that of the proposed HHAOA but could not outperform it because of the premature convergence towards reaching the objective function. In the fixed-dimension multimodal functions (F14–F21), the proposed HHAOA continued the first position by effectively finding the best minimum value. In these functions, AOA has the slowest convergence rate. Surprisingly, the HHO had the least performance and lost the ability to relocate the position to find the desired search regions within the desired number of iterations.

As the number of dimensions increases, the quality of outcomes and the efficiency of alternative approaches deteriorate noticeably. This indicates that HHO can effectively uphold a favorable equilibrium between exploration and exploitation behaviors in scenarios involving multiple variables. The performances in the hybrid functions (F22–F30) have a similar results trend. Notably, in F24, MFO has the best convergence performance, followed by the HHAOA. The proposed HHAOA performs best in non-zero global minima functions by converging nearer to the global minima with fewer iterations, even at less than 50 iterations in most of the functions. Lastly, the proposed HHAOA can deliver exceptional outcomes across all dimensions and consistently outperforms other methods when dealing with problems involving many variables.

#### 4.1.3. Quantitative Analysis

In this section, the efficiency of each algorithm was evaluated by measuring how closely the statistical data match the global minimum of the benchmark function being tested. HHAOA performs better than other algorithms, as it has significant performance outcomes. However, this also means that HHAOA can identify a better optimal solution with fewer iterations and is closer to the objective function, as evidenced by its lower mean value. In contrast, a lower standard deviation (Stdv) signifies more excellent convergence stability, effectiveness, and reliability. Consequently, HHAOA is capable of avoiding local optima with great success. Table 6 show the numerical comparison for all the optimization techniques. Here, the abbreviated terms are Func.—Benchmark Functions, $G_{m}$—Global minima, and Std.Dev—Standard deviation.

The unimodal function results show that the conventional HHO had a lead in reaching the global minima value more than the other algorithms. Surprisingly, GWO and WOA have an average performance compared to the newly developed AOA and SCA algorithms. The proposed HHAOA has notable performances in F4–F7 due to the arithmetic operators’ and hawks’ ability to narrow down the desired global minima and avoid further searching for the exploration locations. Meanwhile, the MFO performs poorly in these functions, which shows its inability to reach even for the singular objective functions.

The HHAOA method is able to reach the global minimum in both multimodal and fixed-dimension functions better than other techniques. HHO reached the desired value in most functions, but HHAOA converged at the exact minimum values in many cases. It’s important to note that almost all algorithms converged at the desired global minimum values in F9 and F16. The MFO competed with the proposed HHAOA in mean and best value in the fixed dimension functions. At the same time, the well-performed GWO and WOA in the unimodal function have a significant setback in this multi-objective function. They struggled to reach the best value in most of the and had an almost massive difference in the final convergence values than the desired global minima.

Lastly, the same results’ trend has been repeated in the hybrid functions. Notably, the HHO and SCA had poor performance and these algorithms quickly progressed in the first few iterations, but then the rate of improvement slowed down. The GWO and MFO had a closer convergence value in most functions, showing the same approach to finding the best optimum values (see Figure 7), whereas the proposed HHAOA had a stable and smooth value closer to the global minima, it suggests that the algorithm converges stably. However, in F23, the HHAOA has a lower standard deviation, indicating that the algorithm consistently finds a better solution for a stable convergence. Overall, these statistical measurements prove that the convergence behavior of the HHAOA helps to identify effective search locations for improving the exploration and exploitation phases of the algorithm.

The Friedman ranking test, which compares algorithms based on their best mean values in numerical analysis, is displayed in Table 7. In this comparative analysis, the Friedman ranking test is used to evaluate the performance of various metaheuristic approaches. The algorithms were ranked according to their mean value for the corresponding functions and the resulting list clearly compares each approach’s effectiveness. The top-performing algorithm is awarded a rank of 1 as it is the closest to the global minimum. On the other hand, the algorithm with the lowest mean value is assigned a rank of 5, indicating its significant deviation from the desired global minimum value. In this analysis, the proposed HHAOA has the smallest average value of 1.967, securing the first rank, which signifies the technique’s effectiveness in identifying the best optimum value with fewer iterations in most benchmark functions. In addition, the HHAOA has a massive 181.342% faster and best mean value compared with the SCA, which has a final average value of 5.534 with the last rank. The HHAOA has 35.587%, 64.412%, and 79.664% of increased performance than the HHO, GWO, and respectively. Lastly, the MFO has an average of 4.2 and secures fifth rank, followed by AOA with the value of 5.067, securing the second-to-last rank.

### 4.2. Simulation Analysis of Industrial WMNs

**Figure 8:**
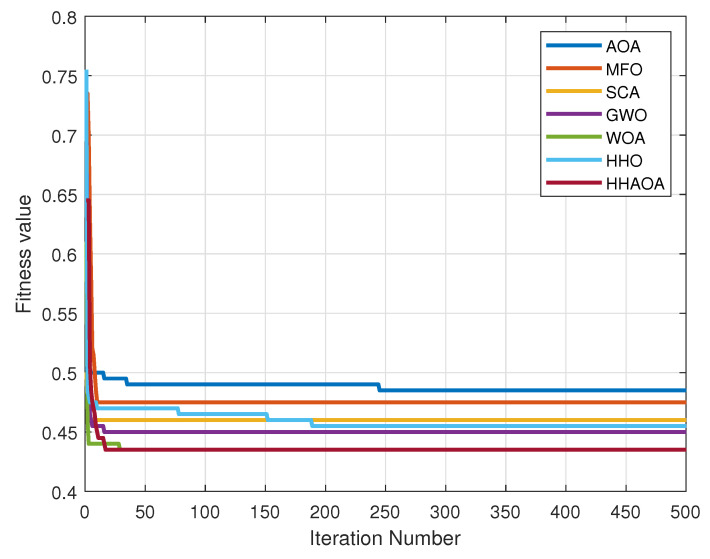
WMN convergence for different algorithms.

Understanding the convergence behavior of optimization algorithms is crucial for obtaining optimal solutions. In the performance evaluation of the proposed HHAOA algorithm against other algorithms, the convergence behavior was analyzed using 500 iterations with 100 runs for 100 search agent sets, shown in Figure 8. Table 8 shows the statistical analysis of the convergence. By observing the convergence ability of these algorithms, their performance can be determined clearly. The objective function was utilized to find the minimum fitness value, which demonstrates improved connectivity and coverage.

In the numerical analysis, the HHAOA method yielded the best outcomes with the least mean value of 0.499. Comparatively, the AOA came in second place with a mean value of 0.531. While the HHAOA method is 6.412% faster than AOA, it is worth noting that the WOA method is lagging with a minimal difference of 0.007. This resulted in WOA reaching a third place with a mean value of 0.456, which is 8.617% higher than the HHAOA method. The GWO, HHO, SCA, and MFO methods were followed in the remaining ranking order, which makes it clear that the proposed HHAOA method produced better results in the numerical analysis.

**Figure 9:**
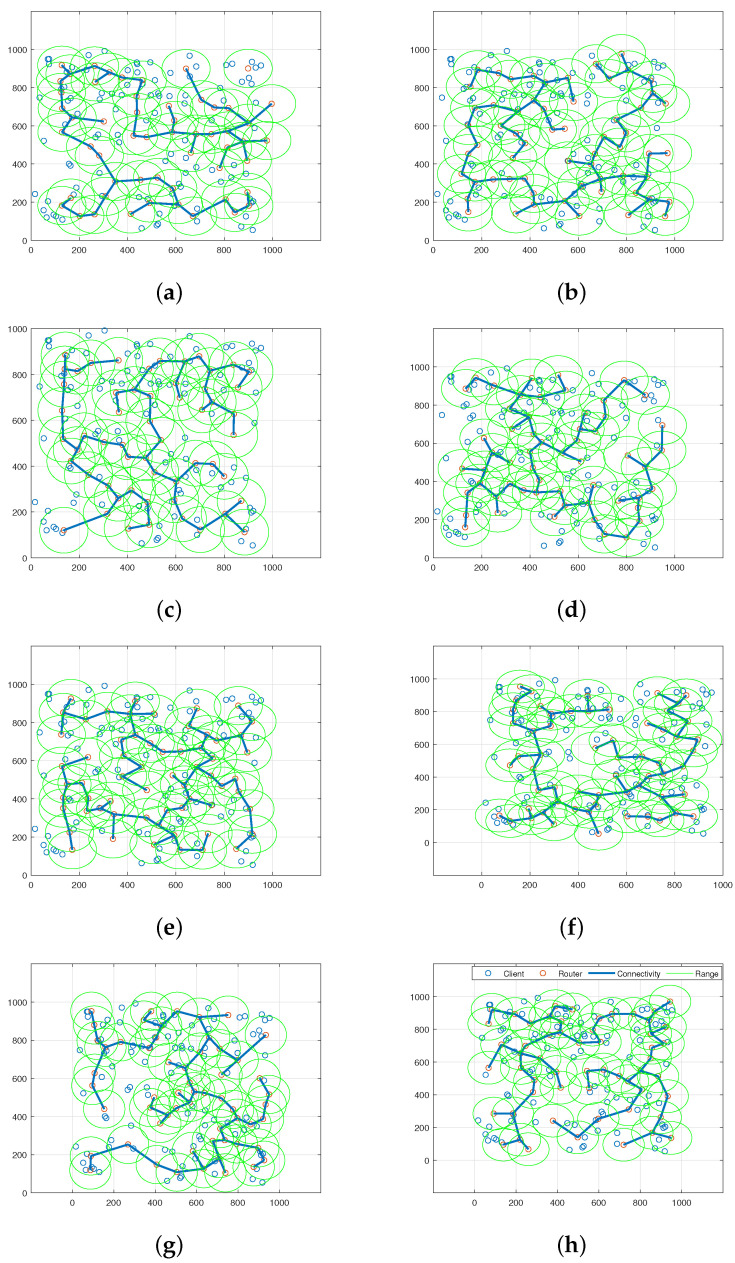
WMN network connectivity and its coverage area for various algorithms. (**a**) Initial Network; (**b**) AOA Optimized WMN; (**c**) MFO Optimized WMN; (**d**) SCA Optimized WMN; (**e**) GWO Optimized WMN; (**f**) WOA Optimized WMN; (**g**) HHO Optimized WMN; (**h**) HHAOA Optimized WMN.

Figure 9 shows the network connectivity and coverage region for the WMN for all the algorithms and the initial WMN topology. Figure 9a illustrates that the initial iteration resulted in insufficient mesh client coverage and high network congestion due to multiple overlapping mesh routers, which increased deployment costs. Even with redundant mesh routers, their connectivity did not reach the maximum capacity of 100%. Figure 9b–h shows the optimized WMN connectivity and coverage of the AOA, MFO, SCA, GWO, WOA, HHO, and the proposed HHAOA, respectively. In these, SCA has the maximum number of disconnected clients and more number of overlapped router placements. It has a 150% less efficient connectivity than the HHO, which secured second place, with four unconnected clients in its optimized WMN. Additionally, the AOA has eight disconnected clients, followed by MFO, WOA, and GWO, with seven, five, and four disconnected clients in the WMN network. However, Figure 9h shows that the proposed HHAOA has dramatically improved the client coverage and optimally deployed the mesh routers to achieve full network connectivity with the least number of routers and had only one disconnected client. Likewise, the proposed HHAOA significantly reduced network congestion by reducing the number of mesh routers by 31.7% while maintaining high coverage and connectivity. Furthermore, other comparing algorithms produced network topologies where mesh routers overlapped, resulting in more significant interference. In contrast, the topology formed by the HHAOA approach was more widely dispersed, resulting in improved client coverage.

### 4.3. Performance Evaluation on Pressure Process Control

**Figure 10:**
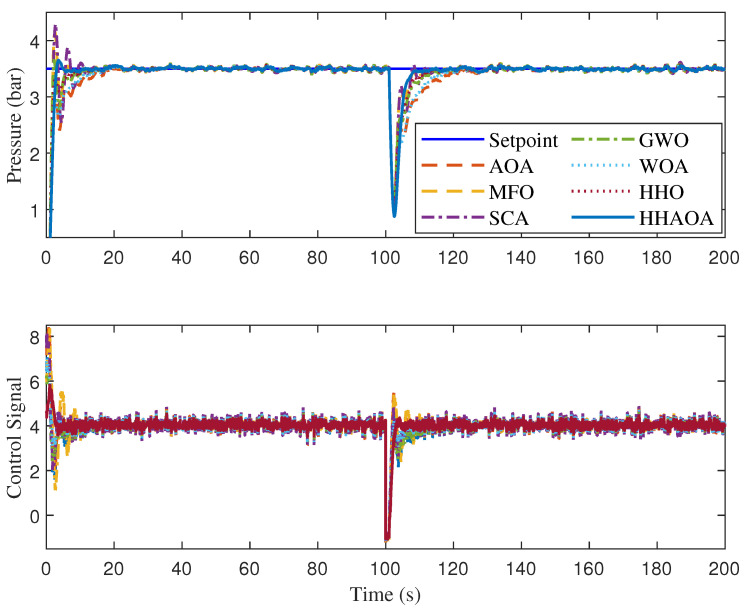
Set-point tracking and disturbance rejection analysis for optimal FOPPI controller.

In the numerical analysis, the comparison was carried out for the process rise time ($t_{r}$), settling time before ($t_{s1}$) disturbance, overshoot (%OS), and settling time after ($t_{s2}$) disturbance injection. In order to evaluate the ability of the FOPPI to decrease the stochastic disturbance and to track the set-point effectively, an external disturbance of 35% was injected at 100 s in the process feedback loop. Table 9 shows the quantitative analysis of the optimized FOPPI controller using different optimization techniques, and the comparative analysis is shown in Figure 10. Here, controller parameters $K_{p}$, $T_{i}$ and λ were obtained based on the ITAE value and integral time was obtained by $T_{i}=\frac{K_{p}}{K_{i}}$, where $K_{i}$ is the integral gain.

The numerical results from the table show that the MFO has the fastest rise time of 0.7552 s, followed by the AOA, HHO, WOA, and SCA with values of 0.8404, 0.8411, and 0.9381 s, respectively. Here the GWO has the slowest rise time of 5.1078 s which is 576.351% slower performance than the fastest MFO. The proposed HHAOA optimized FOPPI had the second-last rise time of 1.4504 s which is almost two times slower than the MFO.

During the settling time before disturbance ($t_{s1}$), even with the slowest rise time, the HHAOA-optimized FOPPI managed to settle faster at 37.2265 s which is 29.7129 s faster than the slowest settled SCA with the settling at 66.9394 s. The result shows that the proposed HHAOA has 79.816% faster performance, which is twice as fast as SCA. The HHO comes second fastest, settling at 49.2073 s, followed by GWO, WOA, MFO, and AOA with the respective values of 52.9052, 54.2561, 57.5289, and 59.6059 s. While observing the settling time $t_{s2}$ after disturbance injection performance, once again, the HHAOA settled faster at 119.2158, which is 34.7982 s ahead of SCA (151.0140s). The same settling time trend is also observed in this, with the HHO settling at 130.0259 s followed by GWO settling at 135.1132 s. The second last rank is for AOA settling at 142.2151 s. In the peak overshoot performance, the HHAOA has 4.7601%, corresponding to a 396.037% reduction of the value, while comparing the SCA at 23.3340%. Amazingly, the GWO has the least peak overshoot value of 2.5131%, followed by WOA, AOA, HHO, and HHAOA, respectively. In this numerical performance analysis, in most of the cases, the Friedman ranking order is observed.

**Figure 11:**
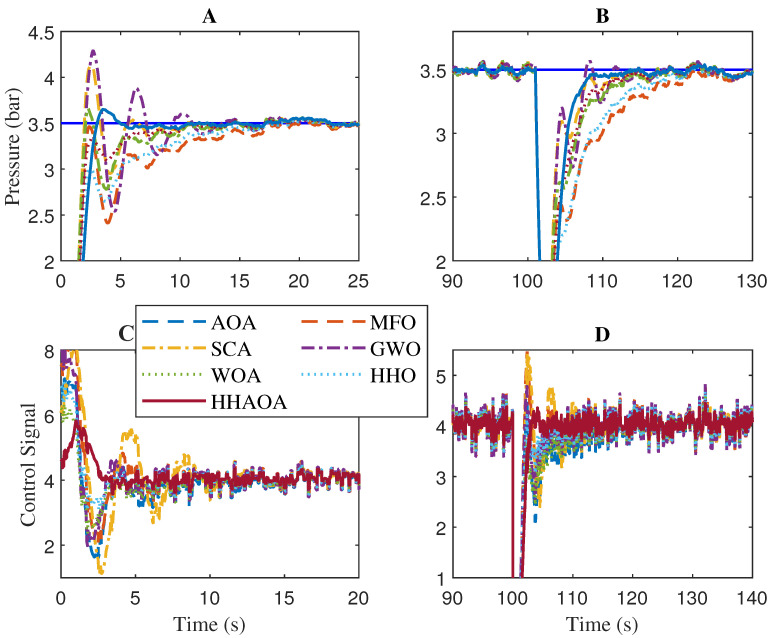
Zoomed view of Figure 10 (**A**) Initial set-point tracking (**B**) Disturbance rejection performance (**C**) Control signal during initial set-point (**D**) Control signal during disturbance rejection.

Based on the comparison results, it is evident that the FOPPI controller optimized by HHAOA is more efficient in producing a stable control signal and rejecting disturbances. However, it is worth noting that the HHAOA may have a slower rise time when tracking the initial set-point, which is noticeable. In the same period, the HHAOA has the fastest settling ahead of others with minimal peak overshoot, are shown in Figure 11, section A. Later, in the external disturbance injection at 100 s, the HHAOA optimized FOPPI had the fastest disturbance recovery and better set-point tracking ability (see, Figure 11, section B). The control actions of the FOPPI for different algorithms are shown in Figure 11, sections C and D. Here, the HHAOA has the robust and effectively generated control signal at 4.3 itself, while others are starting at above 6.0. This is also true in the case of the after-disturbance injection scenario. It is clear from the results that finding the optimized controller parameters for the real-time process plant is essential in order to improve its performance.

## 5. Summary and Conclusions

This section presents the overall summary, which includes the proposed techniques and their advancements given in the first part. Later, the future scope of the current research and the concluding remarks are given.

### 5.1. Summary

This study analyzed different optimization techniques: AOA, MFO, SCA, GWO, WOA, HHO, and the proposed HHAOA. The tests were conducted on various benchmark functions and optimal router placement and connectivity for WMN benchmark functions. Additionally, the optimized FOPPI controller for a real-time pressure process plant was examined. The HHAOA technique outperformed all other algorithms in every comparison analysis, including the rigorous Friedman ranking test. This statistical evidence unequivocally proves the superiority of the HHAOA method over existing techniques. Moreover, it is crucial to highlight the notable contributions that further emphasize the effectiveness of the proposed technique.

1. Utilizing a range of multi-hopping methodologies, the proposed HHAOA can effectively detect the global minima with fewer attempts throughout most benchmark functions, resulting in a more efficient and accurate optimization process. These can be evidenced by its consistently higher mean, best, and standard deviation scores in benchmark functions testing.
2. Through various multi-hopping techniques, the proposed HHAOA is able to identify the global minima in significantly fewer attempts in most of the benchmark functions.
3. In the WMN, the HHAOA demonstrated a highly competent desired objective searching technique, producing the most optimal path for routing network traffic. The algorithm significantly reduces congestion by minimizing the data transmitted across the network, resulting in improved network performance.
4. HHAOA carefully selects the appropriate access points to connect to the network, ensuring that clients are always connected to nearby routers. This makes it an essential optimization technique for successfully deploying wireless mesh networks for the best performance.
5. Notable improvements in producing the robust and smoother control signal of the pressure process were achieved by optimizing the FOPPI controller parameters using HHAOA. These include smaller peak overshoot, dynamic set-point tracking, and practical disturbance rejection ability.

### 5.2. Conclusions

This research article introduced a novel optimization technique called hybrid HHAOA, which combines two existing algorithms, HHO and AOA, to achieve a better performance. In order to assess the efficacy of the HHAOA algorithm, tests were conducted on a total of 33 optimization benchmark functions. The analysis involved a comparison of performance results, which were based on various measures, including mean, global best, worst, and standard deviation. The convergence performance of the HHAOA algorithm is faster at achieving global minima with fewer iterations than other algorithms. The comparison results were evaluated using Friedman ranking, which showed that the proposed HHAOA algorithm significantly outperforms various algorithms with a 181.342% increased performance ranking in terms of the final mean value. In addition, the best connectivity, network overlapping minimization, and optimal router placement for WMN using the proposed HHAOA were simulated for 500 iterations with 100 search agents. The HHAOA produced the most satisfactory desired performance by creating the best client router connectivity with only one client disconnected in the network. Additionally, the network overlapping was significantly reduced, with a 31.7% reduction in the WMN routers substantially minimizing the operational cost. Experimentation was conducted on a real-time pressure process to further demonstrate the HHAOA algorithm’s effectiveness. The findings showed that the proposed algorithm performed best in the dynamic processes. Furthermore, using HHAOA-optimized FOPPI resulted in a more reliable, smooth, and robust control signal, leading to quicker settling and reduced peak overshoot which, in turn, significantly minimizes the wear and tear on the control valve. As part of future research, newer evolutionary algorithms with different mathematical operators will be investigated to enhance the algorithm’s performance. Additionally, attempts will be made to hybridize the HHAOA algorithm with other metaheuristic optimization algorithms to widen its applicability to more complex, real-time industrial and engineering problems.

## Figures and Tables

**Table 1:** Summary of different modifications in the HHO technique.

Ref.	Technique	Application	Improvements	Performance Metrics	Comparing Techniques	Results
[30]	RLHHO	Complex engineering problems	Enhancement of the exploration and exploitation	23 standard functions and 30 CEC 2014 test problems	HHO, DE, PSO, WOA, ABC, GOA, SSA, ALO, BLPSO, PPPSO, SADE, JDE, HHO-DE, DHHO/M	Good stability and prediction accuracy than other algorithms
[38]	HHO-JOS	Engineering design problems	Balancing of the exploration and exploitation phase	30 functions of CEC 2014 29 functions of CEC 2017	HHO, HHO-DO, HHO-SLO, HHO-SO	Good search space identification, better ability to escape from local optima
[31]	Binary HHO, Quadratic Binary BHO	Engineering problems	Binary variables with the feature selection improves phase transition	22 benchmark datasets	BDE, BFPA, BMVO, BSSA, GA	High classification accuracy with improved feature selection
[32]	multi-objective HHO	Real-world problems	New discrete operators for enhancing the hunting technique	13 benchmark datasets and COVID-19 patient datasets	GWO, ABC, GSA, TLBO, BOA	Higher prediction accuracy up to 11.5% with 83.8% better feature selection
[33]	MHHO	Real-world problems	Reconfigured switching techniques for each phases	9 × 9 photovoltaic array	TCT, GA, PSO, GOA	Excellent photovoltaic array reconfiguration with 12.5% on energy saving
[34]	Long-Term Memory HHO	Complex engineering problems	Enhances the diversity of search agents until search termination	10 benchmark functions and Optimal power flow problem	HHO, PSO, ABC, FA, EHO, TEO, BSDE, GOA, GWO	Up to 68% superior performance increase, minimized fuel cost, power loss and emission
[35]	HHO	Large-scale wireless sensor network	None	WSN nodes	EC, PSO, FPA, GWO, SCA, MVO, WOA	Improved network topology for for sink node placement
[36]	Hybrid HH-SS	Wireless sensor networks	A novel fitness function to control the balance between phases	WSN nodes	EAD, EASER, SEAR	Enhanced the packet delivery of 98% with 0.1 s delay
[37]	HHO	Controller tuning	None	DC motor control	HHO, GWO, SFS, IWO,	Improved steady-state error, rise time, overshoot, and settling time.
[39]	MOHHO, HMOHHO	Multi-objective Controller tuning	Hybrid strategies to promote global searching capability	7 test functions and hydraulic turbine governing system	NSGA-III, MOPSO, MOGWO	Better performance during varying load operating conditions

**Table 2:** Summary of different modifications of the AOA technique.

Ref.	Technique	Application	Improvements	Performance Metrics	Comparing Techniques	Results
[42]	MOAOA	Complex engineering problems	Non-dominated sorting technique and maintains the diversity among the obtained best values	35 Real-World Multi-objective Problems and 5 unconstrained problems	NSGWO, MOMVO, MOALO, MOSMA	Best coverage, computational cost, with high efficiency in problem solving
[43]	IANN-AOA, IANN-BCMO	Engineering problems	None	FGM metal and ceramic materials	AOA, BCMO	Improved damage prediction with high accuracy and better damage quantification
[46]	DAOA	Real-world problems	Improved the convergence ability of AOA with better balancing between the phases	Nature and CT COVID-19 images	AO, WOA, SSA, AOA, PSO, MPA, DE	Improved results than comparing techniques, effective-segmented images, PSNR, and SSIM values
[47]	DESMAOA	Complex engineering problems	Random contraction, subtraction and addition strategies to expand the search regions to increase accuracy	23 benchmark functions and 3 engineering problems	SMA, AOA, GWO, WOA, SSA, MVO, PSO	Outperforms other optimization algorithms in terms of speed and accuracy with better local minima switching
[48]	AOA	MPC Controller tuning	Reconfigured switching techniques for each phases	Automatic voltage regulator	ABC, FSA, MOEO, NSGA-II	Good performance in minimizing voltage maximum overshoot and settling time
[49]	IAOA	Complex engineering problems	Forced switching mechanism to avoid local minima trapping	23 benchmark functions and 10 CEC2020 test functions	PSO, SCA, GWO, WOA, SSA, MVO	Better optimization performance in terms of speed and accuracy with better local minima switching
[50]	nAOA	Real-world engineering applications	Distributive mathematical operators to enhance the performance	30 benchmark functions and 3 engineering problems	SA, SCA, GWO, AOA, CPSOGSA, GSA	Better performance during different test problems by position relocation
[51]	AOA	Fuzzy-PID Controller tuning	None	Interconnected power system	TBLO, AOA	Desired performance in variable load, uncertain and offers better contingency
[52]	dAOA	Real-world engineering applications	Chaotic theory to improve the local minima relocation and convergence speed	Proton exchange membrane fuel cell	COA, ALO, WOA, AOA	Reliable and accurate with less training error for the developed machine learning model
[53]	AOAGA	Real-world engineering and complex datasets	Hybridizing GA to improve the searching abilities without compromising on algorithm speed	Several benchmark and two real-world problems	SMA, HHO, SA, MVO, SSA, MFO, GOA, PSO, GWO	Provided a good balance between the number of selected features and classification accuracy

**Table 3:** Different HHAOA conditions and their respective cases in exploration and exploitation phases.

Phase	Technique	Primary Layer	Secondary Layer	Remark
Exploration		|E|≥1 & q≥0.5	c≥0.5	Condition 1 of Equation (1)
		|E|≥1 & q≥0.5	c<0.5	Condition 2 of Equation (1)
		|E|≥1 & q<0.5		Condition 3 of Equation (1)
Exploitation	Soft besiege	|E|<1 & |E|≥0.5 & r≥ 0.5	c<0.5	Condition 1 of Equation (5)
		|E|<1 & |E|≥0.5 & r≥0.5	c≥0.5	Condition 2 of Equation (5)
	Soft besiege- rapid dives	|E|<1 & |E|≥0.5 & r<0.5	c<0.5 or c≥0.5	Condition 1, case 1 or 2 of Equation (7)
		|E|<1 & |E|≥0.5 & r<0.5	c<0.5 or c≥0.5	Condition 2, case 3 or 4 of Equation (7)
	Hard besiege	|E|<1 & |E|<0.5 & r≥0.5	c<0.5	Condition 1 of Equation (8)
		|E|<1 & |E|<0.5 & r≥0.5	c≥0.5	Condition 2 of Equation (8)
	Hard besiege- rapid dives	|E|<1 & |E|<0.5 & r<0.5	c<0.5 or c≥0.5	Condition 1, case 1 or 2 of Equation (9)
		|E|<1 & |E|<0.5 & r<0.5	c<0.5 or c≥0.5	Condition 2, case 3 or 4 of Equation (9)

**Table 4:** Optimization test functions—Unimodal and Multimodal benchmark functions.

Cat.	$G_{m}$	Func.	Description	Range
Unimodal	0	F1	$F\left(x\right)=\sum_{i=1}^{n}x_{i}^{2}$	[100,100]
	0	F2	$F\left(x\right)=\sum_{i=1}^{n}\left|x_{i}\right|+\prod_{i=1}^{n}\left|x_{i}\right|$	[10,10]
	0	F3	$F\left(x\right)=\sum_{i=1}^{d}{\left(\sum_{j=1}^{i}x_{j}\right)}^{2}$	[100,100]
	0	F4	$F\left(x\right)=\max_{i}|X_{i}|,1\leq i\leq n$	[100,100]
	0	F5	$F\left(x\right)=\sum_{i=1}^{n-1}\left[100\left(x_{i+1}^{2}-2x_{i+1}x_{i}^{2}+x_{i}^{4}\right)+\left(x_{i}^{2}-2x_{i}+1\right)\right]$	[30,30]
	0	F6	$F\left(x\right)=\sum_{i=1}^{n}\left(x_{i}^{2}+0.25+x_{i}\right)$	[100,100]
	0	F7	$F\left(x\right)=\sum_{i=1}^{n}ix_{i}^{4}+\mathrm{random}\;\left(0,1\right)$	[128,128]
Multimodal	−148.9829 × n	F8	$F\left(x\right)=\sum_{i=1}^{n}-x_{i}\sin\left(\sqrt{\left|x_{i}\right|}\right)$	[500,500]
	0	F9	$f_{9}\left(x\right)=\sum_{i=1}^{n}\left[x_{i}^{2}-10\cos\left(2\pi x_{i}\right)+10\right]$	[5.12,5.12]
	0	F10	$\begin{array}{c} F\left(x\right)=e+20\left(1+\exp\left(-0.2\sqrt{\frac{1}{n}\sum_{i=1}^{n}x_{i}^{2}}-\left(\frac{1}{n}\sum_{i=1}^{n}\cos\left(2\pi x_{i}\right)\right)\right)\right) \end{array}$	[32,32]
	0	F11	$F\left(x\right)=\frac{1}{4000}\sum_{i=1}^{n}x_{i}^{2}-\prod_{i=1}^{n}\cos\left(\frac{x_{i}}{\sqrt{i}}\right)+1$	[600,600]
	0	F12	$\begin{array}{cc} F(x)= & \frac{\pi }{n}\left\{10\sin\left(\pi y_{1}\right)\right\}+\sum_{i=1}^{n-1}{\left(y_{i}-1\right)}^{2}[1+10\sin^{2}\left(\pi y_{i+1}\right)+\sum_{i=1}^{n}u\left(x_{i},10,100,4\right),\\ & \mathrm{where}\;y_{i}=1+\frac{x_{i}+1}{4},u\left(x_{i},a,k,m\right)\left\{\begin{array}{cc} K{\left(x_{i}-a\right)}^{m} & x_{i}>a\\ 0 & -a\leq x_{i}\geq a\\ K{\left(-x_{i}-a\right)}^{m} & -a\leq x_{i} \end{array}\right. \end{array}$	[50,50]
	0	F13	$\begin{array}{cc} F(x)= & 0.1[\sin^{2}\left(3\pi x_{1}\right)+\sum_{i=1}^{n}{\left(x_{i}-1\right)}^{2}\left[1+\sin^{2}\left(3\pi x_{i}+1\right)\right]+\left(x_{n}^{2}-2x_{n}+1\right)\\ & \left[1+\sin^{2}\left(2\pi x_{n}\right)\right]]+\sum_{i=1}^{n}u\left(x_{i},5,100,4\right) \end{array}$	[50,50]

**Table 5:** Optimization test functions—Fixed dimension multimodal benchmark functions.

Cat.	$G_{m}$	Func.	Description	Range
Fixed-dimension multimodal	1	F14	$F\left(x\right)={\left(\frac{1}{500}+\sum_{j=1}^{25}\frac{1}{j+\sum_{i=1}^{2}{\left(x_{i}-a_{ij}\right)}^{6}}\right)}^{-1}$	[−65,65]
	0.0003	F15	$F\left(x\right)=\sum_{i=1}^{11}{\left[a_{i}-\frac{x_{1}\left(b_{i}^{2}+b_{i}x_{2}\right)}{b_{i}^{2}+b_{i}x_{3}+x_{4}}\right]}^{2}$	[−5,5]
	−1.0316	F16	$4x_{1}^{2}-2.1x_{1}^{4}+\frac{1}{3}x_{1}^{6}+x_{1}x_{2}-4x_{2}^{2}+4x_{2}^{4}$	[−5,5]
	0.398	F17	$F\left(x\right)={\left(x_{2}-\frac{5.1}{4\pi^{2}}x_{1}^{2}+\frac{5}{\pi }x_{1}-6\right)}^{2}+10\left(1-\frac{1}{8\pi }\right)\cos x_{1}+10$	[−5,5]
	3	F18	$\begin{array}{cc} F(x)= & \left[1+{\left(x_{1}+x_{2}+1\right)}^{2}\left(19-14x_{1}+3x_{1}^{2}-14x_{2}+6x_{1}x_{2}+3x_{2}^{2}\right)\right]\\ & \times \left[30+{\left(2x_{1}-3x_{2}\right)}^{2}\times \left(18-32x_{1}+12x_{1}^{2}+48x_{2}-36x_{1}x_{2}+27x_{2}^{2}\right)\right] \end{array}$	[−2,2]
	−3.86	F19	$F\left(x\right)=\sum_{i=1}^{4}c_{i}\exp\left(-\sum_{j=1}^{3}a_{ij}\left(x_{j}^{2}-2p_{ij}x_{j}+p_{ij}^{2}\right)\right)$	[1,3]
	−3.32	F20	$F\left(x\right)=-\sum_{i=1}^{4}c_{i}\exp\left(-\sum_{j=1}^{6}a_{ij}{\left(x_{j}^{2}-2p_{ij}x_{j}+p_{ij}^{2}\right)}^{2}\right)$	[−0,1]
	−1.01532	F21	$F\left(x\right)=-\sum_{i=1}^{5}{\left[\left(X-a_{i}\right){\left(X-a_{i}\right)}^{T}+c_{i}\right]}^{-1}$	[0,10]
Hybrid	−19.2085	F22	$F\left(x\right)=\left|\sin\left(x_{1}\right)\cos\left(x_{2}\right)\exp\left(\left|1-\frac{\sqrt{x_{1}^{2}+x_{2}^{2}}}{\pi }\right|\right)\right|$	[−10,10]
	−117.49797	F23	$F\left(x\right)=\frac{1}{2}\sum_{i=1}^{d}\left(x_{i}^{4}-16x_{i}^{2}+5x_{i}\right)$	[−5,5]
	0	F24	$\begin{array}{c} F\left(x\right)=\sin^{2}\left(\pi w_{1}\right)+\sum_{i=1}^{d-1}{\left(w_{i}-1\right)}^{2}\left[1+10\sin^{2}\left(\pi w_{i}+1\right)\right]+{\left(w_{d}-1\right)}^{2}\left[1+\sin^{2}\left(2\pi w_{d}\right)\right],\\ \;\mathrm{where}\;,w_{i}=1+\frac{x_{i}-1}{4},\;\mathrm{for}\;\mathrm{all}\;i=1,\ldots ,d \end{array}$	[−10,10]
	0	F25	$\begin{array}{c} F\left(x\right)=100{\left(x_{1}^{2}-x_{2}\right)}^{2}+{\left(x_{1}-1\right)}^{2}+{\left(x_{3}-1\right)}^{2}+90{\left(x_{3}^{2}-x_{4}\right)}^{2}\\ +10.1\left({\left(x_{2}-1\right)}^{2}+{\left(x_{4}-1\right)}^{2}\right)+19.8\left(x_{2}-1\right)\left(x_{4}-1\right) \end{array}$	[−10,10]
	−3.86278	F26	$\begin{array}{c} F\left(x\right)=-\sum_{i=1}^{4}\alpha_{i}\exp\left(-\sum_{j=1}^{3}A_{ij}{\left(x_{j}-P_{ij}\right)}^{2}\right),\;\mathrm{where},\;\alpha ={\left(1.0,1.2,3.0,3.2\right)}^{T}\\ A=\left(\begin{array}{ccc} 3.0 & 10 & 30\\ 0.1 & 10 & 35\\ 3.0 & 10 & 30\\ 0.1 & 10 & 35 \end{array}\right)\;P=10^{-4}\left(\begin{array}{ccc} 3689 & 1170 & 2673\\ 4699 & 4387 & 7470\\ 1091 & 8732 & 5547\\ 381 & 5743 & 8828 \end{array}\right) \end{array}$	[0,1]
	−10.5364	F27	$\begin{array}{c} F\left(x\right)=-\sum_{i=1}^{m}{\left(\sum_{j=1}^{4}{\left(x_{j}-C_{ji}\right)}^{2}+\beta_{i}\right)}^{-1},\\ \;\mathrm{where},\;m=10;\beta =\frac{1}{10}{\left(1,2,2,4,4,6,3,7,5,5\right)}^{T}\\ \;C=\left(\begin{array}{cccccccccc} 4.0 & 1.0 & 8.0 & 6.0 & 3.0 & 2.0 & 5.0 & 8.0 & 6.0 & 7.0\\ 4.0 & 1.0 & 8.0 & 6.0 & 7.0 & 9.0 & 3.0 & 1.0 & 2.0 & 3.6\\ 4.0 & 1.0 & 8.0 & 6.0 & 3.0 & 2.0 & 5.0 & 8.0 & 6.0 & 7.0\\ 4.0 & 1.0 & 8.0 & 6.0 & 7.0 & 9.0 & 3.0 & 1.0 & 2.0 & 3.6 \end{array}\right) \end{array}$	[0,10]
	−3.32237	F28	$\begin{array}{c} F\left(x\right)=-\sum_{i=1}^{4}\alpha_{i}\exp\left(-\sum_{j=1}^{6}A_{ij}{\left(x_{j}-P_{ij}\right)}^{2}\right),\\ \;\mathrm{where},\;\alpha ={\left(1.0,1.2,3.0,3.2\right)}^{T}\\ A=\left(\begin{array}{cccccc} 10 & 3 & 17 & 3.50 & 1.7 & 8\\ 0.05 & 10 & 17 & 0.1 & 8 & 14\\ 3 & 3.5 & 1.7 & 10 & 17 & 8\\ 17 & 8 & 0.05 & 10 & 0.1 & 14 \end{array}\right)\\ P=10^{-4}\left(\begin{array}{cccccc} 1312 & 1696 & 5569 & 124 & 8283 & 5886\\ 2329 & 4135 & 8307 & 3736 & 1004 & 9991\\ 2348 & 1451 & 3522 & 2883 & 3047 & 6650\\ 4047 & 8828 & 8732 & 5743 & 1091 & 381 \end{array}\right) \end{array}$	[0,10]
	−9.66015	F29	$F\left(x\right)=-\sum_{i=1}^{d}\sin\left(x_{i}\right)\sin^{2m}\left(\frac{ix_{i}^{2}}{\pi }\right)$	[0,π]
	0	F30	$F\left(x\right)={\left(x_{1}-1\right)}^{2}+\sum_{i=2}^{d}{\left(2x_{i}^{2}-x_{i-1}\right)}^{2}$	[−10,10]

**Table 6:** Quantitative analysis of the different benchmark functions for all the optimization algorithms.

Func.	$G_{m}$	Statistics	AOA	MFO	SCA	GWO	WOA	HHO	HHAOA
F1	1	Mean	1.20 $\times \;10^{-14}$	2.00 $\times \;10^{3}$	2.4136	2.05 $\times \;10^{-33}$	5.28 $\times \;10^{-85}$	9.02 $\times \;10^{-96}$	3.16 $\times \;10^{-6}$
		Best	1.40 $\times \;10^{-270}$	0.2900	0.0036	6.88 $\times \;10^{-35}$	1.47 $\times \;10^{-96}$	1.75 $\times \;10^{-121}$	6.84 $\times \;10^{-17}$
		Worst	6.00 $\times \;10^{-13}$	2.00 $\times \;10^{4}$	27.5418	1.10 $\times \;10^{-32}$	1.62 $\times \;10^{-83}$	4.51 $\times \;10^{-94}$	1.99 $\times \;10^{-12}$
		Std. Dev	8.49 $\times \;10^{-14}$	4.95 $\times \;10^{3}$	4.3477	2.80 $\times \;10^{-33}$	2.34 $\times \;10^{-84}$	2.34 $\times \;10^{-84}$	5.19 $\times \;10^{-13}$
F2	0	Mean	0.0	31.5216	0.0100	6.27 $\times \;10^{-20}$	4.56 $\times \;10^{-53}$	1.89 $\times \;10^{-50}$	1.68 $\times \;10^{-20}$
		Best	0.0	0.1102	9.06 $\times \;10^{-5}$	1.06 $\times \;10^{-20}$	1.07 $\times \;10^{-61}$	4.45 $\times \;10^{-65}$	2.13 $\times \;10^{-9}$
		Worst	0.0	80.0085	0.0740	2.05 $\times \;10^{-19}$	1.32 $\times \;10^{-51}$	9.29 $\times \;10^{-49}$	7.75 $\times \;10^{-7}$
		Std. Dev	0.0	21.6905	0.0140	4.19 $\times \;10^{-20}$	2.20 $\times \;10^{-52}$	1.31 $\times \;10^{-49}$	1.71 $\times \;10^{-7}$
F3	0	Mean	0.0055	1.99 $\times \;10^{4}$	6.44 $\times \;10^{3}$	3.42 $\times \;10^{-8}$	2.75 $\times \;10^{4}$	5.34 $\times \;10^{-81}$	1.09 $\times \;10^{-7}$
		Best	3.63 $\times \;10^{-224}$	1.73 $\times \;10^{3}$	381.3511	3.48 $\times \;10^{-12}$	3.72 $\times \;10^{3}$	3.05 $\times \;10^{-103}$	4.51 $\times \;10^{-13}$
		Worst	0.0711	4.71 $\times \;10^{4}$	2.33 $\times \;10^{4}$	4.48 $\times \;10^{-7}$	4.97 $\times \;10^{4}$	1.85 $\times \;10^{-79}$	2.06 $\times \;10^{-6}$
		Std. Dev	0.0138	1.27 $\times \;10^{4}$	5.13 $\times \;10^{3}$	8.42 $\times \;10^{-8}$	1.11 $\times \;10^{4}$	2.75 $\times \;10^{-80}$	3.61 $\times \;10^{-7}$
F4	0	Mean	0.0221	56.8052	25.9687	2.23 $\times \;10^{-8}$	40.4751	3.71 $\times \;10^{-51}$	7.87 $\times \;10^{-8}$
		Best	7.89 $\times \;10^{-95}$	31.0704	4.4921	2.17 $\times \;10^{-9}$	9.20 $\times \;10^{-5}$	1.68 $\times \;10^{-59}$	0.0
		Worst	0.0514	77.9037	58.7352	1.37 $\times \;10^{-7}$	8.80 $\times \;10^{1}$	6.46 $\times \;10^{-50}$	3.62 $\times \;10^{-7}$
		Std. Dev	0.0201	10.3480	1.23 $\times \;10^{1}$	2.37 $\times \;10^{-8}$	28.5894	1.25 $\times \;10^{-50}$	8.83 $\times \;10^{-8}$
F5	0	Mean	28.2947	1.36 $\times \;10^{4}$	3.17 $\times \;10^{4}$	26.985	27.3725	0.0067	5.22 $\times \;10^{-4}$
		Best	26.9909	133.2200	38.2476	25.2216	26.6853	1.47 $\times \;10^{-5}$	3.08 $\times \;10^{-12}$
		Worst	28.9331	9.04 $\times \;10^{4}$	6.42 $\times \;10^{5}$	28.5425	28.7346	0.0465	0.0088
		Std. Dev	0.4033	3.12 $\times \;10^{4}$	1.12 $\times \;10^{5}$	0.7227	0.4473	0.0086	0.0018
F6	0	Mean	2.8830	797.8558	9.8129	0.4537	0.0811	4.67 $\times \;10^{-5}$	1.78 $\times \;10^{-6}$
		Best	2.2110	0.2982	4.0532	3.75 $\times \;10^{-5}$	0.0124	8.73 $\times \;10^{-8}$	1.65 $\times \;10^{-10}$
		Worst	3.5479	1.01 $\times \;10^{4}$	61.0512	1.2572	0.3644	3.50 $\times \;10^{-4}$	3.41 $\times \;10^{-5}$
		Std. Dev	0.2956	2.73 $\times \;10^{3}$	10.1503	0.3654	0.0844	7.22E-05	5.43 $\times \;10^{-6}$
F7	0	Mean	3.29 $\times \;10^{-5}$	3.3920	0.0682	0.0012	0.0023	1.03 $\times \;10^{-4}$	5.78 $\times \;10^{-5}$
		Best	2.34 $\times \;10^{-7}$	0.0592	0.0023	2.11 $\times \;10^{-4}$	8.20 $\times \;10^{-5}$	1.16 $\times \;10^{-6}$	1.34 $\times \;10^{-7}$
		Worst	1.41 $\times \;10^{-4}$	32.3393	0.4079	0.0031	0.0092	8.79 $\times \;10^{-4}$	3.78 $\times \;10^{-4}$
		Std. Dev	3.40 $\times \;10^{-5}$	7.2993	0.0741	6.13 $\times \;10^{-4}$	0.0021	1.49 $\times \;10^{-4}$	6.68 $\times \;10^{-5}$
F8	−418.982 × n	Mean	−5.59 $\times \;10^{3}$	−8.80 $\times \;10^{3}$	−3.84 $\times \;10^{3}$	−6.27 $\times \;10^{3}$	−1.09 $\times \;10^{4}$	−1.26 $\times \;10^{4}$	−1.25 $\times \;10^{4}$
		Best	−6.81 $\times \;10^{3}$	−1.06 $\times \;10^{4}$	−4.43 $\times \;10^{3}$	−7.77 $\times \;10^{3}$	−1.26 $\times \;10^{4}$	−1.26 $\times \;10^{4}$	−1.26 $\times \;10^{4}$
		Worst	−4.43 $\times \;10^{3}$	−7.08 $\times \;10^{3}$	−3.15 $\times \;10^{3}$	−4.66 $\times \;10^{3}$	−7.61 $\times \;10^{3}$	−1.26 $\times \;10^{4}$	−1.26 $\times \;10^{4}$
		Std. Dev	445.5937	836.3389	236.3415	600.7231	1.70 $\times \;10^{3}$	0.2895	0.1582
F9	0	Mean	0.0075	150.3165	34.7350	1.5057	0.0	0.0	0.0
		Best	0.0	50.8095	0.0348	0.0	0.0	0.0	0.0
		Worst	0.0	244.1201	124.7699	16.4049	0.0	0.0	0.0
		Std. Dev	0.0	43.4749	29.2383	3.6694	0.0	0.0	0.0
F10	0	Mean	8.88 $\times \;10^{-16}$	13.2023	12.8245	4.27 $\times \;10^{-14}$	4.30 $\times \;10^{-15}$	8.88 $\times \;10^{-16}$	7.25 $\times \;10^{-8}$
		Best	8.88 $\times \;10^{-16}$	0.4113	0.0237	3.64 $\times \;10^{-14}$	8.88 $\times \;10^{-16}$	8.88 $\times \;10^{-16}$	1.42 $\times \;10^{-10}$
		Worst	8.88 $\times \;10^{-16}$	19.9599	20.3176	5.42 $\times \;10^{-14}$	7.99 $\times \;10^{-15}$	8.88 $\times \;10^{-16}$	2.91 $\times \;10^{-7}$
		Std. Dev	0.0	8.1829	9.1160	3.84 $\times \;10^{-15}$	2.27 $\times \;10^{-15}$	0.0	7.46 $\times \;10^{-8}$
F11	0	Mean	0.1010	24.1859	0.8524	0.0028	0.0122	1.96 $\times \;10^{-11}$	0.0
		Best	0.0027	0.3535	0.0323	0.0	0.0	4.44 $\times \;10^{-14}$	0.0
		Worst	0.2423	91.0085	1.5062	0.0274	0.2028	4.39 $\times \;10^{-10}$	0.0
		Std. Dev	0.0722	39.8384	0.2655	0.0062	0.0427	7.02 $\times \;10^{-11}$	0.0
F12	0	Mean	0.4309	4.6051	2.77 $\times \;10^{3}$	0.0298	0.0168	2.52 $\times \;10^{-6}$	2.24 $\times \;10^{-8}$
		Best	0.3170	0.3583	0.559	0.0033	0.0012	5.46 $\times \;10^{-9}$	2.08 $\times \;10^{-13}$
		Worst	0.5359	17.6617	1.02 $\times \;10^{5}$	0.0731	0.1415	1.55 $\times \;10^{-5}$	7.07 $\times \;10^{-7}$
		Std. Dev	0.0482	3.5177	1.49 $\times \;10^{4}$	0.0128	0.0274	3.41 $\times \;10^{-6}$	1.01 $\times \;10^{-7}$
F13	0	Mean	2.8075	9.5337	1.09 $\times \;10^{4}$	0.3805	0.2106	2.72 $\times \;10^{-5}$	1.85 $\times \;10^{-6}$
		Best	2.4792	0.6747	2.3552	9.01 $\times \;10^{-5}$	0.0188	7.41 $\times \;10^{-7}$	4.10 $\times \;10^{-13}$
		Worst	2.9930	28.3836	1.48 $\times \;10^{5}$	0.7516	0.8096	1.64 $\times \;10^{-4}$	3.97 $\times \;10^{-5}$
		Std. Dev	0.1253	6.0884	3.28 $\times \;10^{4}$	0.1971	0.1581	3.85 $\times \;10^{-5}$	6.47 $\times \;10^{-6}$
F14	1	Mean	8.9976	1.5926	1.514	3.5875	1.7886	1.1578	0.998
		Best	0.9980	0.9980	0.998	0.998	0.998	0.998	0.998
		Worst	12.6705	4.9500	2.9821	12.6705	10.7632	1.992	0.998
		Std. Dev	4.2660	1.0769	0.8791	3.6212	1.5727	0.3678	0.0
F15	0.0003	Mean	0.0115	9.08 $\times \;10^{-4}$	9.91 $\times \;10^{-4}$	0.0035	5.47 $\times \;10^{-4}$	3.29 $\times \;10^{-4}$	3.45 $\times \;10^{-4}$
		Best	3.59 $\times \;10^{-4}$	3.15 $\times \;10^{-4}$	3.33 $\times \;10^{-4}$	3.07 $\times \;10^{-4}$	3.08 $\times \;10^{-4}$	3.08 $\times \;10^{-4}$	3.08 $\times \;10^{-4}$
		Worst	0.1171	0.0015	0.0016	0.0204	0.0014	4.01 $\times \;10^{-4}$	4.35 $\times \;10^{-4}$
		Std. Dev	0.0244	2.89 $\times \;10^{-4}$	3.69 $\times \;10^{-4}$	0.0074	2.55 $\times \;10^{-4}$	2.24 $\times \;10^{-5}$	3.17 $\times \;10^{-5}$
F16	−1.0316	Mean	−1.0316	−1.0316	−1.0316	−1.0316	−1.0316	−1.0316	−1.0316
		Best	−1.0316	−1.0316	−1.0316	−1.0316	−1.0316	−1.0316	−1.0316
		Worst	−1.0316	−1.0316	−1.0316	−1.0316	−1.0316	−1.0316	−1.0316
		Std. Dev	8.21 $\times \;10^{-8}$	0.0	0.0	0.0	0.0	0.0	0.0
F17	0.398	Mean	0.4080	0.3979	0.3993	0.3979	0.3979	0.3979	0.398
		Best	0.3983	0.3979	0.3979	0.3979	0.3979	0.3979	0.3980
		Worst	0.4434	0.3979	0.4062	0.3981	0.3979	0.3979	0.398
		Std. Dev	0.0089	0.0	0.0016	2.86 $\times \;10^{-5}$	0.0	0.0	0.0
F18	3	Mean	8.4006	3.0	3.0	3.0	3.0	3.0	3.0
		Best	3.0	3.0	3.0	3.0	3.0	3.0	3.0
		Worst	30.0	3.0	3.0	3.0001	3.0001	3.0	3.0
		Std. Dev	10.9094	0.0	0.0	1.43 $\times \;10^{-5}$	2.03 $\times \;10^{-5}$	0.0	0.0
F19	−3.86	Mean	−3.8536	−3.8628	−3.8548	−3.8615	−3.8587	−3.862	−3.86
		Best	−3.8626	−3.8628	−3.8613	−3.8628	−3.8628	−3.8628	−3.86
		Worst	−3.8405	−3.8628	−3.8512	−3.8549	−3.8277	−3.8552	−3.86
		Std. Dev	0.0037	0.0	0.0024	0.0026	0.0065	0.0014	0.0
F20	−3.32	Mean	−3.0815	−3.2168	−3.006	−3.2532	−3.2412	−3.1334	−3.32
		Best	−3.1835	−3.3220	−3.1714	−3.322	−3.3219	−3.311	−3.32
		Worst	−2.8472	−3.1376	−2.5881	−3.0604	−3.0807	−2.8095	−3.32
		Std. Dev	0.0675	0.0448	0.1305	0.077	0.0915	0.0976	0.0
F21	−10.153	Mean	1.20 $\times \;10^{-14}$	2.00 $\times \;10^{3}$	2.4136	2.05 $\times \;10^{-33}$	5.28 $\times \;10^{-85}$	9.02 $\times \;10^{-96}$	3.16 $\times \;10^{-16}$
		Best	1.40 $\times \;10^{-270}$	0.2900	0.0036	6.88 $\times \;10^{-35}$	1.47 $\times \;10^{-96}$	1.75 $\times \;10^{-121}$	6.84 $\times \;10^{-17}$
		Worst	6.00 $\times \;10^{-13}$	2.00 $\times \;10^{4}$	27.5418	1.10 $\times \;10^{-32}$	1.62 $\times \;10^{-83}$	4.51 $\times \;10^{-94}$	1.99 $\times \;10^{-12}$
		Std. Dev	8.49 $\times \;10^{-14}$	4.95 $\times \;10^{3}$	4.3477	2.80 $\times \;10^{-33}$	2.34 $\times \;10^{-84}$	2.34 $\times \;10^{-84}$	5.19 $\times \;10^{-13}$
F22	−19.2085	Mean	0.0	31.5216	0.0100	6.27 $\times \;10^{-20}$	4.56 $\times \;10^{-53}$	1.89 $\times \;10^{-50}$	1.68 $\times \;10^{-20}$
		Best	0.0	0.1102	9.06 $\times \;10^{-5}$	1.06 $\times \;10^{-20}$	1.07 $\times \;10^{-61}$	4.45 $\times \;10^{-65}$	2.13 $\times \;10^{-9}$
		Worst	0.0	80.0085	0.0740	2.05 $\times \;10^{-19}$	1.32 $\times \;10^{-51}$	9.29 $\times \;10^{-49}$	7.75 $\times \;10^{-7}$
		Std. Dev	0.0	21.6905	0.0140	4.19 $\times \;10^{-20}$	2.20 $\times \;10^{-52}$	1.31 $\times \;10^{-49}$	1.71 $\times \;10^{-7}$
F23	−117.497	Mean	0.0055	1.99 $\times \;10^{4}$	6.44 $\times \;10^{3}$	3.42 $\times \;10^{-8}$	2.75 $\times \;10^{4}$	5.34 $\times \;10^{-81}$	1.09 $\times \;10^{-7}$
		Best	3.63 $\times \;10^{-224}$	1.73 $\times \;10^{3}$	381.3511	3.48 $\times \;10^{-12}$	3.72 $\times \;10^{3}$	3.05 $\times \;10^{-103}$	4.51 $\times \;10^{-13}$
		Worst	0.0711	4.71 $\times \;10^{4}$	2.33 $\times \;10^{4}$	4.48 $\times \;10^{-7}$	4.97 $\times \;10^{4}$	1.85 $\times \;10^{-79}$	2.06 $\times \;10^{-6}$
		Std. Dev	0.0138	1.27 $\times \;10^{4}$	5.13 $\times \;10^{3}$	8.42 $\times \;10^{-8}$	1.11 $\times \;10^{4}$	2.75 $\times \;10^{-80}$	3.61 $\times \;10^{-7}$
F24	0	Mean	0.0221	56.8052	25.9687	2.23 $\times \;10^{-8}$	40.4751	3.71 $\times \;10^{-51}$	7.87 $\times \;10^{-8}$
		Best	7.89 $\times \;10^{-95}$	31.0704	4.4921	2.17 $\times \;10^{-9}$	9.20 $\times \;10^{-5}$	1.68 $\times \;10^{-59}$	0.0
		Worst	0.0514	77.9037	58.7352	1.37 $\times \;10^{-7}$	8.80 $\times \;10^{1}$	6.46 $\times \;10^{-50}$	3.62 $\times \;10^{-7}$
		Std. Dev	0.0201	10.3480	1.23 $\times \;10^{1}$	2.37 $\times \;10^{-8}$	28.5894	1.25 $\times \;10^{-50}$	8.83 $\times \;10^{-8}$
F25	0	Mean	28.2947	1.36 $\times \;10^{4}$	3.17 $\times \;10^{4}$	26.985	27.3725	0.0067	5.22 $\times \;10^{-4}$
		Best	26.9909	133.2200	38.2476	25.2216	26.6853	1.47 $\times \;10^{-5}$	3.08 $\times \;10^{-12}$
		Worst	28.9331	9.04 $\times \;10^{4}$	6.42 $\times \;10^{5}$	28.5425	28.7346	0.0465	0.0088
		Std. Dev	0.4033	3.12 $\times \;10^{4}$	1.12 $\times \;10^{5}$	0.7227	0.4473	0.0086	0.0018
F26	−3.8628	Mean	2.8830	797.8558	9.8129	0.4537	0.0811	4.67 $\times \;10^{-5}$	1.78 $\times \;10^{-6}$
		Best	2.2110	0.2982	4.0532	3.75 $\times \;10^{-5}$	0.0124	8.73 $\times \;10^{-8}$	1.65 $\times \;10^{-10}$
		Worst	3.5479	1.01 $\times \;10^{4}$	61.0512	1.2572	0.3644	3.50 $\times \;10^{-4}$	3.41 $\times \;10^{-5}$
		Std. Dev	0.2956	2.73 $\times \;10^{3}$	10.1503	0.3654	0.0844	7.22 $\times \;10^{-5}$	5.43 $\times \;10^{-6}$
F27	−10.5364	Mean	3.29 $\times \;10^{-5}$	3.3920	0.0682	0.0012	0.0023	1.03 $\times \;10^{-4}$	5.78 $\times \;10^{-5}$
		Best	2.34 $\times \;10^{-7}$	0.0592	0.0023	2.11 $\times \;10^{-4}$	8.20 × 10^−5^	1.16 $\times \;10^{-6}$	1.34 $\times \;10^{-7}$
		Worst	1.41 $\times \;10^{-4}$	32.3393	0.4079	0.0031	0.0092	8.79 $\times \;10^{-4}$	3.78 $\times \;10^{-4}$
		Std. Dev	3.40 $\times \;10^{-5}$	7.2993	0.0741	6.13 $\times \;10^{-4}$	0.0021	1.49 $\times \;10^{-4}$	6.68 $\times \;10^{-5}$
F28	−3.3224	Mean	−5.59 $\times \;10^{3}$	−8.80 $\times \;10^{3}$	−3.84 $\times \;10^{3}$	−6.27 $\times \;10^{3}$	−1.09 $\times \;10^{4}$	−1.26 $\times \;10^{4}$	−1.25 $\times \;10^{2}$
		Best	−6.81 $\times \;10^{3}$	−1.06 $\times \;10^{4}$	−4.43 $\times \;10^{3}$	−7.77 $\times \;10^{3}$	−1.26 $\times \;10^{4}$	−1.26 $\times \;10^{4}$	−1.26 $\times \;10^{4}$
		Worst	−4.43 $\times \;10^{3}$	−7.08 $\times \;10^{3}$	−3.15 $\times \;10^{3}$	−4.66 $\times \;10^{3}$	−7.61 $\times \;10^{3}$	−1.26 $\times \;10^{4}$	−1.26 $\times \;10^{4}$
		Std. Dev	445.5937	836.3389	236.3415	600.7231	1.70 $\times \;10^{3}$	0.2895	0.1582
F29	−9.6602	Mean	0.0075	150.3165	34.7350	1.5057	0.0	0.0	0.0
		Best	0.0	50.8095	0.0348	0.0	0.0	0.0	0.0
		Worst	0.0	244.1201	124.7699	16.4049	0.0	0.0	0.0
		Std. Dev	0.0	43.4749	29.2383	3.6694	0.0	0.0	0.0
F30	0	Mean	8.88 $\times \;10^{-16}$	13.2023	12.8245	4.27 $\times \;10^{-14}$	4.30 $\times \;10^{-15}$	8.88 $\times \;10^{-16}$	7.25 $\times \;10^{-8}$
		Best	8.88 $\times \;10^{-16}$	0.4113	0.0237	3.64 $\times \;10^{-14}$	8.88 $\times \;10^{-16}$	8.88 $\times \;10^{-16}$	1.42 $\times \;10^{-10}$
		Worst	8.88 $\times \;10^{-16}$	19.9599	20.3176	5.42 $\times \;10^{-14}$	7.99 $\times \;10^{-15}$	8.88 $\times \;10^{-16}$	2.91 $\times \;10^{-7}$
		Std. Dev	0.0	8.1829	9.1160	3.84 $\times \;10^{-15}$	2.27 $\times \;10^{-15}$	0.0	7.46 $\times \;10^{-8}$

**Table 7:** Different benchmark functions of Friedman ranking test for the comparing algorithms.

Function	AOA	MFO	SCA	GWO	WOA	HHO	HHAOA
1	5	7	6	3	2	1	4
2	1	7	6	5	2	3	4
3	4	6	5	2	7	1	3
4	4	7	5	2	6	1	3
5	5	6	7	3	4	2	1
6	5	7	6	4	3	2	1
7	1	7	6	4	5	3	2
8	3	5	2	4	6	7	1
9	4	7	6	5	3	2	1
10	1	7	6	4	3	1	5
11	5	7	6	3	4	2	1
12	5	6	7	4	3	2	1
13	5	6	7	4	3	2	1
14	7	4	3	6	5	2	1
15	7	4	5	6	3	1	2
16	1	1	1	1	1	1	1
17	7	1	6	1	1	1	5
18	7	1	1	1	1	1	1
19	7	1	6	3	5	2	4
20	6	4	7	2	3	5	1
21	6	4	7	2	3	5	1
22	7	1	6	1	1	1	1
23	6	1	5	7	1	3	4
24	7	1	6	3	5	4	2
25	3	4	6	5	7	2	1
26	7	2	6	4	5	3	1
27	7	4	6	1	3	5	2
28	6	4	7	2	3	5	1
29	7	3	6	2	4	5	1
30	6	1	7	3	4	5	2
**Average**	**5.067**	**4.2**	**5.534**	**3.234**	**3.534**	**2.667**	**1.967**
**Final Rank**	**6**	**5**	**7**	**3**	**4**	**2**	**1**

**Table 8:** Convergence analysis for the WMN for all the algorithms.

Optimization	Mean	Best	Worst	Std. Dev
AOA	0.531	0.485	0.673	0.0858
MFO	0.488	0.475	0.735	0.0841
SCA	0.472	0.46	0.524	0.0675
GWO	0.463	0.450	0.720	0.0587
WOA	0.456	0.435	0.634	0.0046
HHO	0.469	0.455	0.755	0.0674
HHAOA	0.449	0.435	0.613	0.0025

**Table 9:** Experimental quantitative analysis of various optimized FOPPI controller on the pressure process.

Optimization Type	$K_{p}$	$K_{i}$	λ	$t_{r}$	$t_{s1}$	$t_{s1}$	%OS
AOA	1.207	0.836	0.97	0.8404	59.6059	142.2151	2.8723
MFO	2.713	1.183	0.98	0.7552	57.5289	140.1284	18.9260
SCA	1.881	1.062	0.96	1.0371	66.9394	151.0140	23.3440
GWO	1.672	0.714	0.96	5.1078	52.9052	135.1132	2.5131
WOA	2.015	0.615	0.99	0.9381	54.2561	137.2006	2.6118
HHO	2.427	1.031	0.99	0.8411	49.2073	130.0259	4.8653
HHAOA	2.359	0.901	0.98	1.4504	37.2265	119.2158	4.7061

## Data Availability

No new data were created or analyzed in this study. Data sharing is not applicable to this article.

## Abbreviations

The following abbreviations are used in this manuscript:

ALO	Ant Lion Optimizer
AOA	Arithmetic Optimization Algorithm
ABC	Artificial Bee Colony
BMVO	Binary Multi-Verse Optimizer
BSSA	Binary Salp Swarm Algorithm
BLPSO	Biogeography-based Learning Particle Swarm Optimization
BOA	Butterfly Optimization Algorithm
DESMAOA	Deep Ensemble of Slime Mold Arithmetic Optimization Algorithm
DE	Differential Evolution
FOPPI	Fractional-order Predictive PI
GA	Genetic Algorithm
GOA	Grasshopper Optimisation Algorithm
GSA	Gravitational Search Algorithm
GWO	Grey Wolf Optimization
HART	Highway Addressable Remote Transducer
HHO	Harris Hawk Optimization
HHSS	Harris Hawk and Salp Swarm
HH-AOA	Harris Hawk-Arithmetic Optimization Algorithm
HMOHHO	Hybrid Mutation Operator Harris Hawks Optimization
IIAOA	mproved Arithmetic Optimization Algorithm
IANN	Improved Artificial Neural Network
ISA	International Society of Automation
JOS	Joint Opposite Selection
MPC	Model Predictive Controller
MHHO	Modified Harris Hawk Optimizer
MFO	Moth Flame Optimization
MOHHO	Multi-Objective Harris Hawks Optimization
MVO	Multi-Verse Optimizer
NCS	Network Control Systems
NGSA	Non-dominated Sorting Genetic Algorithm
PPPSO	Predator Prey Particle Swarm Optimizations
SSA	Salp Swarm Algorithm
SADE	Self Adaptive Differential Evolution
SCA	Sine Cosine Algorithm
TLBO	Teaching Learning Based Optimization
WOA	Whale Optimization Algorithm
WMN	Wireless Mesh Networks
WIA-PA	Wireless network for Industrial Automation-Process Automation
